# Deciphering the Clinical Significance and Kinase Functions of GSK3α in Colon Cancer by Proteomics and Phosphoproteomics

**DOI:** 10.1016/j.mcpro.2023.100545

**Published:** 2023-04-08

**Authors:** Li Gao, Ying Lu, Hai-Ning Chen, Zhigui Li, Meng Hu, Rou Zhang, Xiuxuan Wang, Zhiqiang Xu, Yanqiu Gong, Rui Wang, Dan Du, Shan Hai, Shuangqing Li, Dan Su, Yuan Li, Heng Xu, Zong-Guang Zhou, Lunzhi Dai

**Affiliations:** 1 National Clinical Research Center for Geriatrics and General Practice Ward/International Medical Center Ward, General Practice Medical Center, State Key Laboratory of Biotherapy, West China Hospital, Sichuan University, Chengdu, China; 2 Colorectal Cancer Center, Department of General Surgery, West China Hospital, Sichuan University, Chengdu, China; 3 Department of Gastrointestinal Surgery, West China Hospital, Sichuan University, Chengdu, China; 4 Advanced Mass Spectrometry Center, Research Core Facility, Frontiers Science Center for Disease-related Molecular Network, West China Hospital, Sichuan University, Chengdu, China; 5 Institute of Digestive Surgery, West China Hospital, Sichuan University, Chengdu, China

**Keywords:** colon cancer, GSK3α, GSK3β, phosphorylation, substrate specificity

## Abstract

GSK3α and GSK3β are two GSK3 isoforms with 84% overall identity and 98% identity in their catalytic domains. GSK3β plays important roles in the pathogenesis of cancer, while GSK3α has long been considered a functionally redundant protein of GSK3β. Few studies have specifically investigated the functions of GSK3α. In this study, unexpectedly, we found that the expression of GSK3α, but not GSK3β, was significantly correlated with the overall survival of colon cancer patients in 4 independent cohorts. To decipher the roles of GSK3α in colon cancer, we profiled the phosphorylation substrates of GSK3α and uncovered 156 phosphosites from 130 proteins specifically regulated by GSK3α. A number of these GSK3α-mediated phosphosites have never been reported before or have been incorrectly identified as substrates of GSK3β. Among them, the levels of HSF1^S303p^, CANX^S583p^, MCM2^S41p^, POGZ^S425p^, SRRM2^T983p^, and PRPF4B^S431p^ were significantly correlated with the overall survival of colon cancer patients. Further pull-down assays identified 23 proteins, such as THRAP3, BCLAF1, and STAU1, showing strong binding affinity to GSK3α. The interaction between THRAP3 and GSK3α was verified by biochemical experiments. Notably, among the 18 phosphosites of THRAP3, phosphorylation at S248, S253, and S682 is specifically mediated by GSK3α. Mutation of S248 to D (S248D), which mimics the effect of phosphorylation, obviously increased cancer cell migration and the binding affinity to proteins related to DNA damage repair. Collectively, this work not only discloses the specific function of GSK3α as a kinase but also suggests GSK3α as a promising therapeutic target for colon cancer.

## Highlights

• High levels of GSK3α predict poor prognosis of colon cancer patients.
• Identification of 156 phosphosites specifically regulated by GSK3α, not GSK3β.
• THRAP3 has been identified as a phospho-substrate of GSK3α.
• GSK3α-mediated phosphorylation of THRAP3 at S248 promotes cancer cell migration.

## Graphical Abstract

**Figure:**
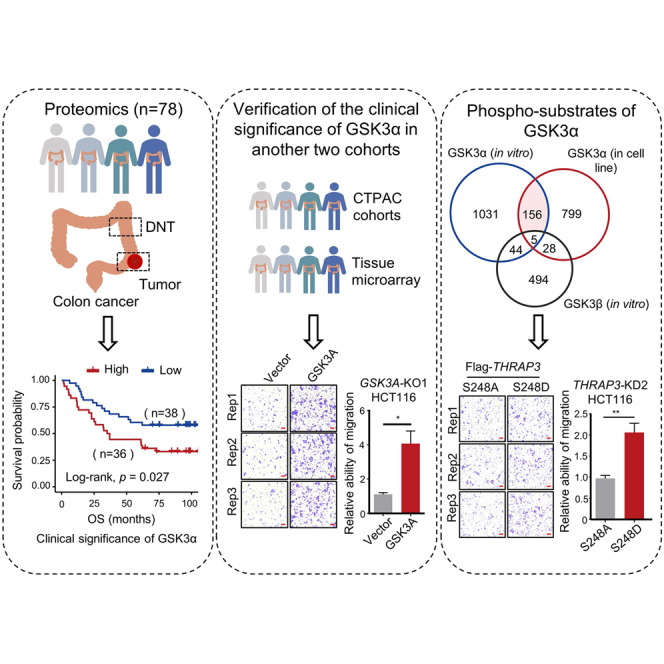

## In Brief

GSK3α and GSK3β are distinct isoforms, with GSK3β studied in cancer development while GSK3α was considered redundant. Yet, our research has revealed that GSK3α, rather than GSK3β, is significantly correlated with colon cancer patients' survival. Integrative *In vitro* and in cell line phosphoproteomics identified 156 phosphosites specifically regulated by GSK3α. Notably, phosphorylation of THRAP3^S248^ remarkably enhanced colon cancer cell migration. These findings suggest that GSK3α may have unique and important roles in colon cancer development and progression.

Colorectal cancer (CRC) is the third most common cancer and has the second highest mortality rate worldwide (1). Distant metastasis is responsible for approximately 90% of CRC deaths (2). Since the first approval of cetuximab and bevacizumab in 2004, a great number of agents targeting the kinases EGFR and VEGFR have entered the clinic (3). Glycogen synthase kinase GSK3 is a key kinase in the Wnt/β–catenin signaling pathway (4). It is responsible for the phosphorylation of β-catenin, which subsequently triggers the ubiquitination and degradation of β-catenin. Adenomatous polyposis coli mutations may inactivate GSK3 and lead to activation of the Wnt/β–catenin signaling pathway, which is an initiation factor of more than 80% of spontaneous CRCs (5). GSK3 is a multitasking molecule and serves as either a tumor promoter or tumor suppressor in cancers (6, 7). To date, in addition to β-catenin (8), more than 40 phospho-substrates of GSK3, such as TSC2 (9), SMAD1 (10), GLI3 (11), SUFU (12), cyclin D1 (13), Snail (14), IκB (15), and NICD1 (16), have been described.

GSK3 has two isoforms, GSK3α and GSK3β, encoded by *GSK3A* and *GSK3B*, respectively. The two GSK3 isoforms have 84% overall identity and 98% identity in their catalytic domains (17). GSK3β can shuttle between the nucleus and cytoplasm, while GSK3α is usually found in the cytoplasm due to its glycine-rich *N* terminus. However, calcium or calpain products can promote GSK3α nuclear accumulation (18). The activities of GSK3α and GSK3β are modulated by their phosphorylation (19, 20). Despite tremendous progress in the study of GSK3 functions, most previous studies focused on GSK3β or failed to distinguish the functional differences between GSK3α and GSK3β. Thus far, only a few studies have specifically examined GSK3α, including the functional elucidation of GSK3α in organ aging (21), ESC differentiation (22), atherosclerosis and hepatic steatosis (23), and hippocampal N-methyl-d-aspartate receptor-dependent long-term depression (24). However, the clinical significance and specific functions of GSK3α as a kinase in cancer have been largely ignored.

In this study, we screened the key kinases that affect the survival of colon cancer patients and may be potential targets. Based on our proteome and immunostaining data and publicly available proteome data from 4 independent colon cancer cohorts (25, 26), we showed that GSK3α, but not GSK3β, was able to predict the prognosis of colon cancer patients. To decipher the roles of GSK3α in colon cancer, we profiled the *in vitro* and cellular phospho-substrates of GSK3α and identified 156 phosphosites specifically regulated by GSK3α but not GSK3β. Further pull-down assays identified 23 proteins, such as THRAP3, BCLAF1, and STAU1, with strong binding affinity specific to GSK3α. Notably, among the 18 phosphosites of THRAP3, phosphorylation at S248, S253, and S682 is specifically mediated by GSK3α *in vitro* and in cell lines. Mutation of S248 to D (S248D) to mimic the effect of its phosphorylation obviously increased the migratory ability of cancer cells and the binding affinity to proteins related to DNA damage repair (DDR).

## Experimental Procedures

### Declaration of Helsinki Ethical Principles

This work was conducted according to the Declaration of Helsinki ethical principles. Paired colon cancer samples from 78 treatment-naïve patients were obtained from West China Hospital. The tissues were quickly frozen in liquid nitrogen after surgery and stored in a −80 °C refrigerator until use. The pathological sections were processed by the Department of Pathology of West China Hospital, a laboratory accredited by the College of American Pathologists. Both colon cancer tissues used for omics and colon cancer tissues used for immunohistochemistry were obtained with informed consent and the approval of the Ethics Committee of Biology Research, West China Hospital at Sichuan University (Permission number: 2020(374)). Clinical information, including age, sex, tumor stage, and follow-up information, was collected.

### Protein Extraction

Tissue and cell samples were lysed in radioimmunoprecipitation assay buffer (50 mM Tris–HCl, 150 mM NaCl, 1% NP-40, 0.5% sodium deoxycholate, protease inhibitor, phosphatase inhibitor, pH = 7.5). Tissue samples were homogenized with a gentleMACS Dissociator (Miltenyi Biotec GmbH) using the procedure “protein 01. 01”. The crude lysates were sonicated (model JY92-IIN, 227.5 W, 3 s on 10 s off, 5 min) and then centrifuged at 20,000*g* and 4 °C for 30 min. The supernatants were collected and the protein concentrations were measured.

### Peptide Digestion and TMT Labeling

A tandem mass tag (TMT)-based strategy was used for quantitative proteomics and phosphoproteomics analysis of tumors and distant normal tissues (DNTs). For proteomics, 78 paired tumors and DNTs were divided into 21 TMT-labeled batches with common reference samples. Fifty micrograms of protein lysates were reduced with 10 mM tris (2-carboxyethyl) phosphine (Sigma) at 56 °C for 1 h and alkylated with 20 mM iodoacetamide in the dark at room temperature for 30 min. Subsequently, the protein lysates were precipitated with methanol/chloroform/water and the precipitate was resuspended in 50 mM triethylammonium bicarbonate (Sigma) and digested with MS sequencing grade trypsin (Promega, 1:50 enzyme-to-substrate ratio) at 37 °C overnight. The trypsin was then inactivated by heating at 95 °C for 2 min. The digested peptides and the common reference sample were labeled by TMT10plex reagents (Thermo Fisher Scientific). After quenching with hydroxylamine, the colon cancer samples and the reference sample in the same batch were combined. The combined peptides were desalted by reversed-phase C18 SPE columns (Phenomenex, 10 mg) and separated into 120 fractions by reversed-phase HPLC (RP-HPLC) at a flow rate of 1 ml/min. These fractions were combined into 15 fractions before MS analysis.

For phosphoproteomics of colon cancer tissues, 70 paired tumors and DNTs were divided into 18 TMT-labeled batches with common reference samples. Forty micrograms of digested peptides from each sample were labeled with TMT reagents. The combined TMT-labeled peptides were separated into 15 fractions by C18 SPE columns (Phenomenex, 100 mg) and then combined into 5 fractions, which were further used for phosphopeptide enrichment.

### Enrichment of Phosphopeptides

PureCube Fe-nitrilotriacetic acid (NTA)-agarose beads were used for phosphopeptide enrichment and purchased from Cube Biotech. The desalted peptides were dissolved in loading buffer (85% acetonitrile (ACN), 0.1% TFA) and then incubated with Fe-NTA agarose beads at 25 °C for 1 h. After incubation, the agarose beads were washed 4 times with washing buffer (80% ACN, 0.1% TFA). Then, the phosphopeptides were eluted from the agarose beads by elution buffer (40% ACN, 15% ammonium hydroxide). The eluted peptides were acidized with 10% TFA and dried. The samples were desalted and further analyzed by LC-MS/MS.

### LC-MS/MS Analysis

For proteomics profiling of colon cancer tissues, desalted peptides of each fraction were analyzed using an EASY-nanoLC 1200 system (Thermo Fisher Scientific) coupled to an Orbitrap Exploris 480 mass spectrometer (Thermo Fisher Scientific). The peptides were separated in the analytical column with a 65 min gradient with 4% to 100% buffer B (80% ACN, 0.1% formic acid) at a flow rate of 300 nl/min. Under the data-dependent acquisition mode, MS spectra were acquired from 350 to 1800 m/z with a resolution of 60,000 at m/z = 200. The normalized automatic gain control (AGC) target (%) was set as 300%. For the MS2 analysis, a 0.7 m/z isolation window and normalized collision energy (NCE) of 36% were selected, with exclusion of precursor ions with charge states of z = 1 or 8 or unassigned charge states.

For phosphoproteomics of colon cancer tissues, the enriched phosphopeptides were analyzed by an EASY-nanoLC 1200 system coupled to a Q Exactive HF-X mass spectrometer. The flow rate of the analytical column was 330 nl/min with a 65 min gradient from 6% to 100% buffer B (80% ACN, 0.1% formic acid). For the full MS scans, ions with m/z ranging from 350 to 1600 were acquired under data-dependent acquisition mode by orbitrap with a resolution of 60,000 at m/z = 200. For the MS2 scans, the top 20 most intense parent ions were selected with an isolation window of 0.6 m/z and fragmented with stepped NCE values of 25% and 31%.

For cellular phosphoproteomics analysis, the enriched phosphopeptides were desalted with ZipTip (Merck Millipore) and analyzed by an EASY-nLC 1200 system coupled to a Q Exactive HF-X mass spectrometer (Thermo Fisher Scientific). The flow rate of the analytical column was 330 nl/min with a 90 min gradient from 12% to 100% buffer B (80% ACN, 0.1% formic acid). For the full MS, ions with m/z ranging from 350 to 1800 m/z were acquired by orbitrap resolution of 60,000 at m/z = 200. For the MS2 scans, the top 20 most intense parent ions were selected with an isolation window of 1.6 m/z and fragmented with stepped NCE values of 25% and 27%. The maximum injection time for MS2 was 64 ms and the dynamic exclusion time was 50 s. For *in vitro* phosphoproteomics analysis, the enriched phosphopeptides were desalted with ZipTip (Merck Millipore) and analyzed by an EASY-nanoLC 1000 coupled to a Q Exactive Plus mass spectrometer (Thermo Fisher Scientific). The flow rate of the analytical column was 330 nl/min with a 65 min gradient from 10% to 90% buffer B (80% ACN, 0.1% formic acid). For the full MS, ions with m/z ranging from 350 to 1800 were acquired by an orbitrap resolution of 60,000 at m/z = 200. For the MS2 scans, the top 20 most intense parent ions were selected for MS2 analysis and fragmented with stepped NCE values of 25% and 27%. The maximum injection time for MS2 was 64 ms and the dynamic exclusion time was 50 s.

For analysis of coimmunoprecipitated proteins, the digested peptides were desalted with ZipTip (Merck Millipore) and analyzed by an EASY-nanoLC 1000 coupled to a Q Exactive Plus mass spectrometer (Thermo Fisher Scientific). Peptides were separated in the analytical column with a 65 min gradient from 10% to 90% buffer B (80% ACN, 0.1% formic acid) at a flow rate of 330 nl/min. For the full MS, the mass range was from 350 to 1800 m/z with a resolution of 60,000 at 200 m/z. The AGC value was set at 3e^6^, with a maximum fill time of 20 ms. For the MS/MS scans, the top 20 most intense parent ions were selected with an isolation window of 1.6 m/z and fragmented with an NCE of 27%. The AGC was set at 1e^6^, with a maximum fill time of 64 ms and a resolution of 17,500.

### MS Data Searching

The proteomics data were analyzed with MaxQuant (version 1.6, https://www.maxquant.org) and searched against the Swiss-Prot human protein sequence database (20,431 protein sequences, updated on 2019/07/16). The proteolytic enzyme was trypsin/p and two missed cleavage sites were allowed. Oxidation of methionine and acetylation of the protein N terminus was set as variable modifications. Cysteine carbamidomethylation was set as a fixed modification. The maximum peptide mass was set to 12,000 Da and the minimum amino acid length was set to 6. The first search mass tolerance was set to 20 ppm and the main search peptide tolerance was set to 4.5 ppm. The mass tolerance for fragment ions was set at 0.5 Da. The phosphoproteomics data were searched with the same database and similar parameters. Phosphorylation at S, T, or Y residues was also set as a variable modification. For label-free phosphoproteomics, the “match between runs” was enabled.

### Bioinformatics and Statistical Analysis

For processing the output datasheets of proteome data of colon cancer tissues, the contaminant and reverse hits were first removed. The proteins with two or more unique peptides were kept. The total protein intensity of each sample as well as the reference sample was then normalized in each batch. The protein intensity of the sample was divided by that of the reference sample in the same batch and then the proteome data of 21 batches were combined. The proteins with missing values in less than 50% of the samples were used for subsequent data analysis. Kaplan–Meier survival curves (log-rank test) and the Cox proportional hazard model for overall survival (OS) analyses were implemented to identify the most important kinases. The above analyses were achieved through the “survival” and “survminer” packages in R. The filter criteria for kinase biomarkers were as follows: log-rank *p* value <0.05, Cox *p* value <0.05 (for samples in all stages) or <0.1 (for samples in each tumor-node-metastasis [TNM] stage), and HR ≥ 2 or ≤0.5.

For processing the output datasheets of phosphoproteome data of colon cancer tissues, the contaminant and reverse hits were first removed. The phosphopeptides with localization probabilities greater than 0.75 were assigned to reliable identification. The total phosphopeptide intensity of each sample in the same batch was normalized to the same level. The phosphopeptide intensity of the sample was divided by that of the reference sample in the same batch and then the phosphoproteome data of 18 batches were combined. The phosphosites with missing values in less than 50% of the samples were used for subsequent data analysis. Kaplan–Meier survival curves (log-rank test) and Cox proportional hazard models for OS analyses were carried out to identify the most important phosphosites. The filter criteria for important phosphosites were as follows: log-rank *p* value <0.05, Cox *p* value <0.05, and HR > 1 or <1. The functions of phosphoproteins were analyzed by Metascape. For processing the output datasheets of phosphoproteome data *in vitro* or in cell lines, the contaminant and reverse hits were first removed. The phosphopeptides with localization probabilities greater than 0.75 were assigned to reliable identification. The phosphosites with ratio (GSK3α/Control) >1.5 and *p* value <0.05 (Student’s *t* test) or only detected in the GSK3α-treated group and *p* value <0.05 (Student’s *t* test) were defined as phosphosites significantly upregulated by GSK3α *in vitro*. Similarly, phosphosites with ratio (*GSK3A*-KO/WT) <0.667 and *p* value <0.05 (Student’s *t* test) or only detected in the control group and *p* value <0.05 (Student’s *t* test) were defined as phosphosites significantly regulated by GSK3α in the cell lines. The functions of phosphoproteins were analyzed by Metascape.

For processing the output table of coimmunoprecipitation proteome data, the contaminant and reverse hits were first removed. Proteins with ≥2 unique peptides were selected for data analysis. Based on the label-free quantification intensities, the quantified proteins with ratio (GSK3α/control) >1.5 or ratio (GSK3β/control) >1.5 (Student’s *t* test, *p* < 0.05) or only identified in the GSK3α or GSK3β overexpression groups (*p* < 0.05, Student’s *t* test) were supposed to be the binding proteins of GSK3α or GSK3β. For the binding proteins of THRAP3 mutants, the proteins with foldchanges >1.5 (Student’s *t* test, *p* < 0.05) or exclusively identified in that group were considered the binding proteins of the corresponding THRAP3 mutant. The functions of binding proteins were analyzed by Metascape.

### Cell Lines and Cell Culture

*The* HEK293T and human colon cancer cell lines HCT116 and RKO were grown in Dulbecco’s modified Eagle’s medium and the DLD-1 cell line was grown in RPMI-1640 medium. The medium was supplemented with 10% fetal bovine serum (FBS) (FSP500, ExCell Bio), 100 U/ml penicillin and 100 μg/ml streptomycin (15,140–122, Gibco).

### Western Blotting Analysis

The protein samples were separated by 10% or 12% SDS-PAGE and transferred to polyvinylidene fluoride membranes (ISEQ00010, Millipore). After blocking with 4% nonfat milk, the polyvinylidene fluoride membranes were incubated with primary antibody (1:2000) at 4 °C overnight and then incubated with HRP secondary antibody (1:10,000) at room temperature for 1 h. Antibodies against the following proteins were used: GAPDH (60004-1-Ig, Proteintech), GSK3α (R1312–1, HUABIO), GSK3β (ET1607–71, HUABIO), Flag (66008-4-Ig, Proteintech), Myc (R1208–1, HUABIO), THRAP3 (19744-1-AP, Proteintech), E-cadherin (ET1607–75, HUABIO), N-cadherin (22018-1-AP, Proteintech), and vimentin (10366-1-AP, Proteintech).

### Measurement of Kinase Activity

Human *GSK3A* (1–483 aa) and *GSK3B* (1–420 aa) were cloned into the pGEX-6P1 vector and transduced into *Escherichia coli* BL21-Gold (DE3) cells. The recombinant proteins GST-GSK3α and GST-GSK3β were extracted by lysis buffer A (50 mM Tris–HCl, 150 mM NaCl, 2.5 mM DTT, 1 mM EGTA, 1 mM EDTA, 1 mM PMSF, 25% glycerol, pH = 7.5), and lysis buffer B (50 mM Na_3_PO_4_, 200 mM NaCl 2 mM DTT, 1 mM PMSF, 25% glycerol, pH = 7.0), respectively. The recombinant proteins were purified by GST affinity resin (GE Healthcare).

The kinase activity was measured by a Kinase-Lumi assay Kit (S0150M, Beyotime). Five micrograms of extracted HCT116 protein as the substrate was mixed with 0, 0.01, 0.02, 0.05, 0.1, 0.2, 0.5, and 1.0 μg of recombinant GST-GSK3α protein. The mixture was added to the kinase reaction buffer (20 mM Tris–HCl, 10 mM MgCl_2_, 5 mM DTT, 25 μM ATP, pH = 7.5) and incubated at 30 °C for 0, 1, 2, or 3 h. The reaction products were mixed with the Kinase-Lumi detection reagent and the fluorescence was detected by Multikinetic microplate reader (Biotech).

### *In Vitro* Kinase Assays

Two hundred micrograms of extracted proteins from HCT116 cells were mixed with the recombinant GST-GSK3α protein and kinase reaction buffer (20 mM Tris–HCl, 10 mM MgCl_2_, 5 mM DTT, and 25 μM ATP, pH = 7.5) and incubated at 30 °C for 2 h. A reaction without recombinant GST-GSK3α was used as a control. The experimental and control assays were repeated three times. The phosphopeptides of each sample were enriched by Fe-NTA agarose beads and analyzed by LC-MS/MS.

### Generation of Stable Colon Cancer Cell Lines

*GSK3A-* or *THRAP3-*knockdown (KD) HCT116 or DLD-1 cell lines were generated using a CRISPR/Cas9 system. There were two guide RNAs targeting the human *GSK3A* gene and two gRNAs targeting the human *THRAP3* gene. The HCT116 and DLD-1 cells after knockdown of the target genes were screened by puromycin (ST551, Beyotime). To obtain *GSK3A*-KO HCT116 cells, a limiting dilution strategy was used to obtain monoclonal cells. The gRNA sequence was as follows:

*GSK3A*-gRNA1-sense: 5′-CACCGGGCGGAAAGGCATCTGTCG-3′

*GSK3A*-gRNA1-antisense: 5′-AAACCGACAGATGCCTTTCCGCCC-3′

*GSK3A*-gRNA2-sense: 5′-CACCGGCACTAGCTTCCCGCCGCC-3′

*GSK3A*-gRNA2-antisense: 5′-AAACGGCGGCGGGAAGCTAGTGCC-3′

*THRAP3*-gRNA1-sense: 5′-CACCGCGAGAGAGAGATCGGCTTC-3′

*THRAP3*-gRNA1-antisense: 5′-AAACGAAGCCGATCTCTCTCTCGC-3′

*THRAP3*-gRNA2-sense: 5′-CACCGCTCTCGTTCAAGGAAGCGC-3′

*THRAP3*-gRNA2-antisense: 5′-AAACGCGCTTCCTTGAACGAGAGC-3′

To reconstruct the *GSK3A* and *THRAP3* mutants in *GSK3A* and *THRAP3*-KD HCT116 cell lines, respectively, we inserted the complementary DNA into a vector (pCDH-CMV-MCS-EF1) and abrogated the binding of gRNAs by altering the protospacer adjacent motif (PAM) sequence with the following primers:

*GSK3A*-PAM1-sense: 5′-AGGCATCTGTCGGAGCCATGGGT-3′

*GSK3A*-PAM1-antisense: 5′-ACCCATGGCTCCGACAGATGCCT-3′

*THRAP3*-PAM2-sense: 5′-TTCAAGGAAGCGCAGACTGAGTTCTAGGT-3′

*THRAP3*-PAM2-antisense: 5′-ACCTAGAACTCAGTCTGCGCTTCCTTGAA-3′

To construct the *THRAP3* mutants, 6 paired primers were used and designed as follows:

*THRAP3*^S248A^-sense: 5′-AGTCCTCGGGAGCGAGCCCCAGCTCTCAAAA-3′

*THRAP3*^S248A^-antisense: 5′-TTTTGAGAGCTGGGGCTCGCTCCCGAGGACT-3′

*THRAP3*^S248D^-sense: 5′-AGTCCTCGGGAGCGAGACCCAGCTCTCAAAA-3′

*THRAP3*^S248D^-antisense: 5′-TTTTGAGAGCTGGGTCTCGCTCCCGAGGACT-3′

*THRAP3*^S253A^-sense: 5′-AGCCCAGCTCTCAAAGCACCCCTCCAGTCTGT-3′

*THRAP3*^S253A^-antisense: 5′-CAGACTGGAGGGGTGCTTTGAGAGCTGGGCT-3′

*THRAP3*^S253D^-sense: 5′-AGCCCAGCTCTCAAAGATCCCCTCCAGTCTGTG-3′

*THRAP3*^S253D^-antisense: 5′-CACAGACTGGAGGGGATCTTTGAGAGCTGGGCT-3′

*THRAP3*^S248/253A^-sense: 5′-AGCGAGCACCAGCTCTCAAAGCACCCCTCCA-3′

*THRAP3*^S248/253A^-antisense: 5′-TGGAGGGGTGCTTTGAGAGCTGGTGCTCGCT-3′

*THRAP3*^S248/253D^-sense: 5′-AGCGAGATCCAGCTCTCAAAGATCCCCTCCA-3′

*THRAP3*^S248/253D^-antisense: 5′-TGGAGGGGATCTTTGAGAGCTGGATCTCGCT-3′

### Colony Formation Assay

For the colony formation assay, empty vector-transduced cells, and *GSK3A*-KD (or KO) cells were seeded into 6-well plates and incubated for approximately 10 days. The cells were fixed with 4% paraformaldehyde at room temperature for 30 min and then stained with 1% (w/v) crystal violet. Colonies were imaged and counted by ImageJ (https://imagej.net).

### Cell Migration Assay

HCT116 cells (2 × 10^5^) or DLD-1 cells (1 × 10^5^) were plated in an 8.0 μm, 24-well cell culture insert (353,097, Corning Life Science) in 200 μl of medium containing 1% FBS serum, with medium containing 10% FBS at the bottom of the insert. The HCT116 and DLD-1 cells were incubated for 24 h and 20 h, respectively. Then, the cells were fixed with 4% paraformaldehyde at room temperature for 30 min and stained with 1% (w/v) crystal violet. The migrated cells were imaged and counted by ImageJ.

### Immunostaining Assays

A colon cancer tissue microarray was obtained from the West China Hospital Biobank of Sichuan University. Immunohistochemistry was performed using an anti-GSK3α antibody (R13121, HUABIO). The expression level of GSK3α in each spot in the microarray was evaluated by Image-Pro Plus 6.0 software (https://www.meyerinst.com/brand/mediacybernetics).

### Coimmunoprecipitation

Cells were harvested 48 h after transfection in cold PBS and lysed with lysis buffer (25 mM Tris–HCl, 150 mM NaCl, 1% NP-40, 5% glycerol and protease inhibitor, phosphatase inhibitor, pH = 7.4) on ice for 30 min and centrifuged at 20,000*g*. GSK3α-Flag, GSK3β-Flag, and Flag-THRAP3 were pulled down with anti-Flag magnetic beads (HY-K0207, MedChemExpress), and THRAP3 proteins were pulled down with an anti-THRAP3 antibody (19744-1-AP, Proteintech). The interacting proteins were eluted by elution buffer (50 mM Tris–HCl, 2% SDS, 10% glycerol, and 1% β-mercaptoethanol, pH = 6.8) and heated at 95 °C for 5 min. The eluted products were analyzed by Western blots or LC-MS/MS.

### Experimental Design and Statistical Rationale

For the proteomics of colon cancer tissues, 156 samples were divided into 21 batches with a common reference sample and 12 quality control (QC) samples were used to monitor the stability of the machine. For the phosphoproteomics of colon cancer tissues, 140 samples were divided into 18 batches with a common reference sample. To ensure strict analysis, proteins quantified in more than 50% of samples with ≥2 unique peptides or phosphosites quantified in more than 50% of samples with localization probability ≥0.75 were used for subsequent data analysis. The significance of differences between groups was calculated by the Wilcoxon test. The significance of survival analysis was calculated by the log-rank test and the best cutoff was applied. For phosphoproteomics of GSK3α *in vitro* or in cell lines, each experiment had 3 replicates. The phosphosites that were identified in at least 4 of 6 samples or only identified in at least 2 of 3 samples in the control or experimental group were used for difference analysis by Student’s *t* test. For the identification of binding proteins of GSK3α-Flag or GSK3β-Flag, each experiment had 3 replicates. The proteins that were identified in at least 4 of 6 samples or only identified in at least 2 of 3 samples in the control or experimental group with ≥2 unique peptides were used for difference analysis by Student’s *t* test. For the identification of binding proteins of THRAP3 mutants (THRAP3^S248A^, THRAP3^S248D^, THRAP3^S253A^, and THRAP3^S253D^), each experiment had 4 replicates. Proteins identified in more than 50% of samples or only identified in the control or experimental group were used for difference analysis by Student’s *t* test.

## Results

### Kinome Analysis Reveals That GSK3α, but Not GSK3β, Correlates With the OS of Colon Cancer Patients

Paired tumors and DNTs collected from 78 treatment-naive colon cancer patients were subjected to TMT-based proteomics (supplemental Fig. S1*A*) (27). The 156 samples were divided into 21 batches with a common reference sample and 12 QC samples were used to monitor the stability of the machine. The correlation coefficients of the 12 QC samples ranged from 0.90 to 1.00 (supplemental Fig. S1*B*). In addition, correlation analysis of the common reference samples in different batches also indicated high reproducibility of the data acquisition (supplemental Fig. S1*C*). Principal component analysis showed that these samples were well separated into DNT and tumor groups (supplemental Fig. S1*D*), without an obvious batch effect (supplemental Fig. S1*E*).

**Fig. 1:**
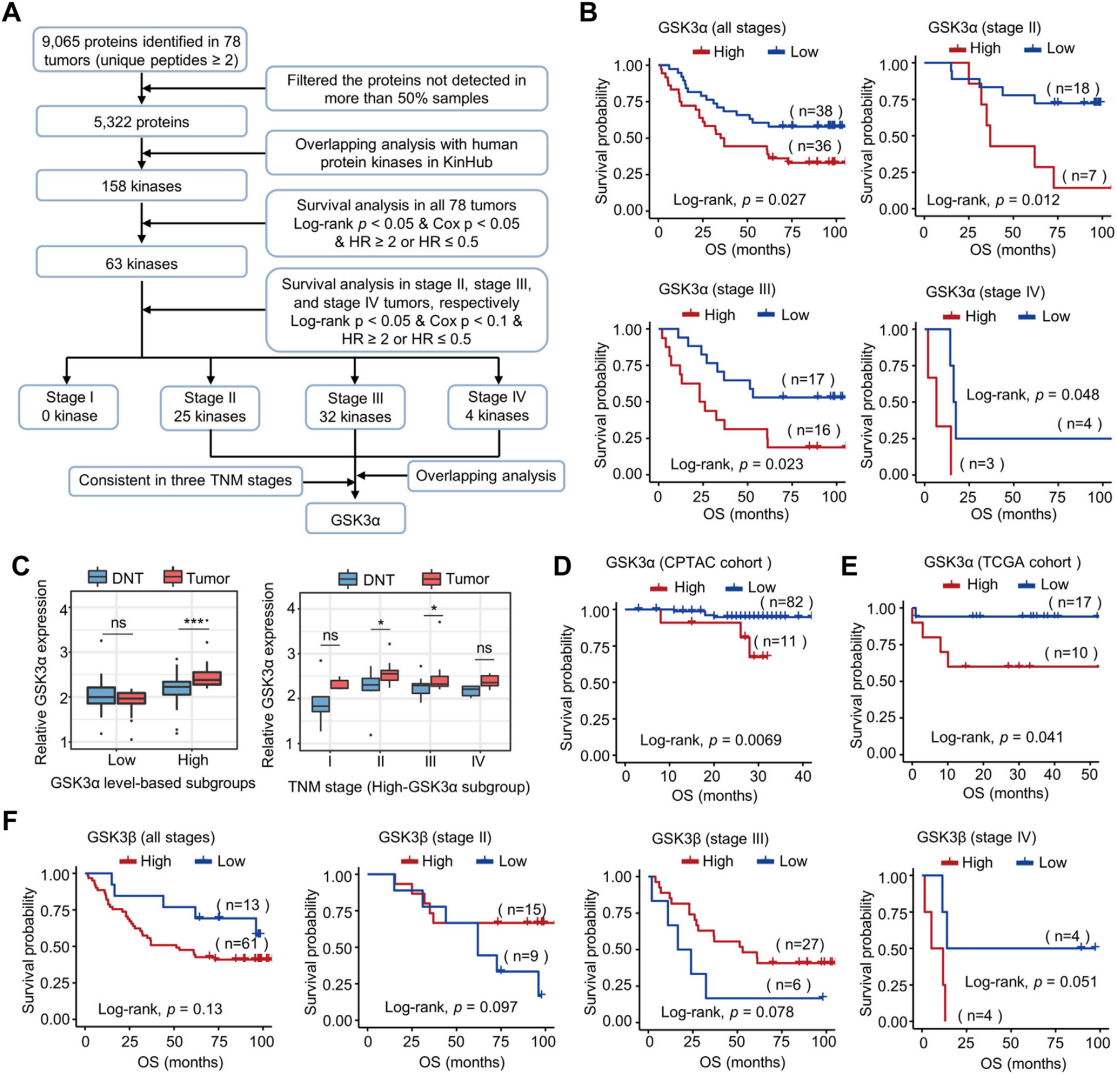
**GSK3α is an important kinase significantly correlated with colon cancer prognosis.***A*, the workflow of screening the critical kinases in colon cancer based on the proteome data. *B*, survival analysis of colon cancer patients with different levels of GSK3α in all tumors or in tumors of different TNM stages (stage II, stage III, and stage IV) based on the proteome data. Of the 78 patients, GSK3α was detected in 74 patients. The *p* value was calculated by the log-rank test and the best cutoff was applied. *C*, relative GSK3α expression between tumors and DNTs in the low-GSK3α and high-GSK3α subgroups and the relative levels of GSK3α in four TNM stages in the high-GSK3α subgroup based on the proteome data. The low-GSK3α and high-GSK3α subgroups were divided based on the best cutoff of OS in Fig. 1*B*. The significance of differences between different groups was calculated by the Wilcoxon test, ∗*p* < 0.05, ∗∗∗*p* < 0.001. *D* and *E*, survival analysis of colon cancer patients with different GSK3α expression levels in tumors based on the proteome data of the CPTAC cohort (*D*) and TCGA cohort (*E*). The *p* value was calculated by the log-rank test and the best cutoff was selected. *F*, survival analysis of colon cancer patients with different levels of GSK3β in all tumors or in tumors of different TNM stages (stage II, stage III, and stage IV) based on the proteome data. The *p* value was calculated by the log-rank test and the best cutoff was applied. OS, overall survival.

Next, we screened the kinases significantly correlated with the OS of colon cancer patients. Out of the 5322 proteins with 2 or more unique peptides that were identified in more than 50% of samples, 158 kinases were identified. Based on Kaplan–Meier survival analysis and Cox proportional hazards regression modeling in all 78 tumors, the levels of 63 kinases were found to be significantly correlated with the prognosis of colon cancer patients. The clinical significance of the 63 kinases was further analyzed separately in the tumors of each TNM stage. As a result, out of the 63 kinases, 0 kinases in stage I tumors, 25 kinases in stage II tumors, 32 kinases in stage III tumors, and 4 kinases in stage IV tumors were found to be significantly associated with the OS of colon cancer patients (supplemental Table S1). Overlapping analysis revealed that only GSK3α and MAP2K1 could predict the prognosis of colon cancer patients in stages II-IV (Fig. 1*A*). However, the hazard ratios of MAP2K1 in stage II-IV patients were inconsistent (supplemental Fig. S2). In contrast, high levels of GSK3α in tumors were significantly positively associated with poor prognosis in colon cancer, whether in 74 tumors of different stages or in tumors of stage II, III, and IV alone (Fig. 1*B*), indicating that GSK3α is a key kinase in colon cancer. Notably, in the tumors of colon cancer patients grouped into high-GSK3α based on the best cutoff (Fig. 1*B*), the expression differences of GSK3α between tumors and DNTs were much more significant (Fig. 1*C*), although some stages were no longer significant after TNM staging, probably due to the limited number of samples. The survival correlation result was confirmed in the Clinical Proteomic Tumor Analysis Consortium (CPTAC) cohort (Fig. 1*D*) and the Cancer Genome Atlas (TCGA) cohort (Fig. 1*E*). Notably, no significant correlation was found between the levels of GSK3β in tumors and the prognosis of colon cancer patients (Fig. 1*F*). Collectively, these results indicate that GSK3α is an important kinase in colon cancer and that high expression of GSK3α predicts unfavorable OS in colon cancer patients.

### Further Validation of the Clinical Significance of GSK3α and Functional Studies

**Fig. 2:**
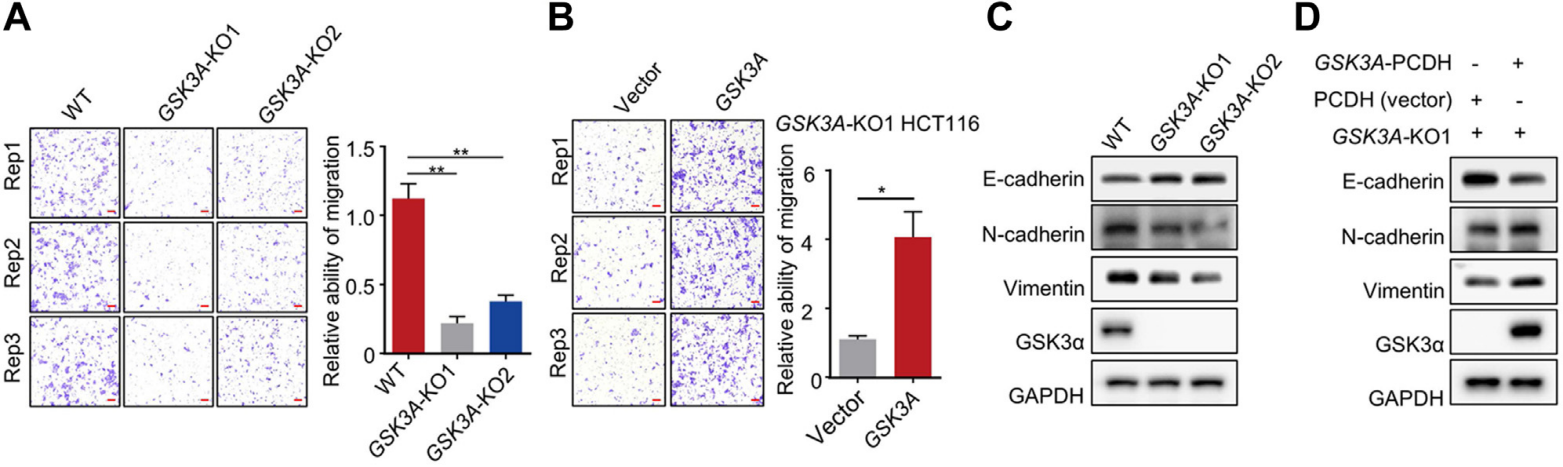
**Effects of GSK3α on cell phenotypes.***A* and *B*, representative images of HCT116 colon cancer cell line migration after *GSK3A*-KO (*A*) and *GSK3A* re-expression (*B*). The scale bar represents 100 μm. Each assay was repeated three times. The significance of differences between different groups was calculated by paired *t* test, ∗*p* < 0.05, ∗∗*p* < 0.01. *C* and *D*, immunoblots of GSK3α, E-cadherin, N-cadherin, and vimentin in HCT116 cells in response to *GSK3A*-KO (*C*) or re-expression (*D*).

To further confirm the clinical significance of GSK3α, we next performed immunohistochemical straining of colon cancer tissue microarrays from another independent colon cancer cohort using a specific anti-GSK3α antibody (supplemental Fig. S3, *A* and *B* and supplemental Table S2). As expected, high levels of GSK3α were also significantly correlated with unfavorable prognosis in colon cancer patients, whether in 111 tumors of different stages or in tumors of stage II, III, and IV alone (supplemental Fig. S3*C*). To reveal the functions of GSK3α, silencing of *GSK3A* in DLD-1 and HCT116 colon cell lines was carried out by a CRISPR/Cas9 system. The colony formation abilities (supplemental Fig. S4, *A* and *B*) and the migratory abilities (Figs. 2*A* and S4*C*) of HCT116 and DLD-1 cells were significantly reduced when *GSK3A* was silenced, while the phenotypes were restored by re-expressing *GSK3A* (Figs. 2*B* and S4*D*). Immunoblotting detection of epithelial-mesenchymal transition markers showed increased E-cadherin and reduced vimentin and N-cadherin expression after *GSK3A* silencing (Figs. 2*C* and S4*E*). The expression of these markers could be recovered by restoring GSK3α expression (Fig. 2*D*), consistent with the observed cell phenotypes. Collectively, these results suggest that the high expression of GSK3α in tumors is disastrous and may promote colon cancer malignancy.

### Identification of *In Vitro* Phospho-Substrates Catalyzed by GSK3α

**Fig. 3:**
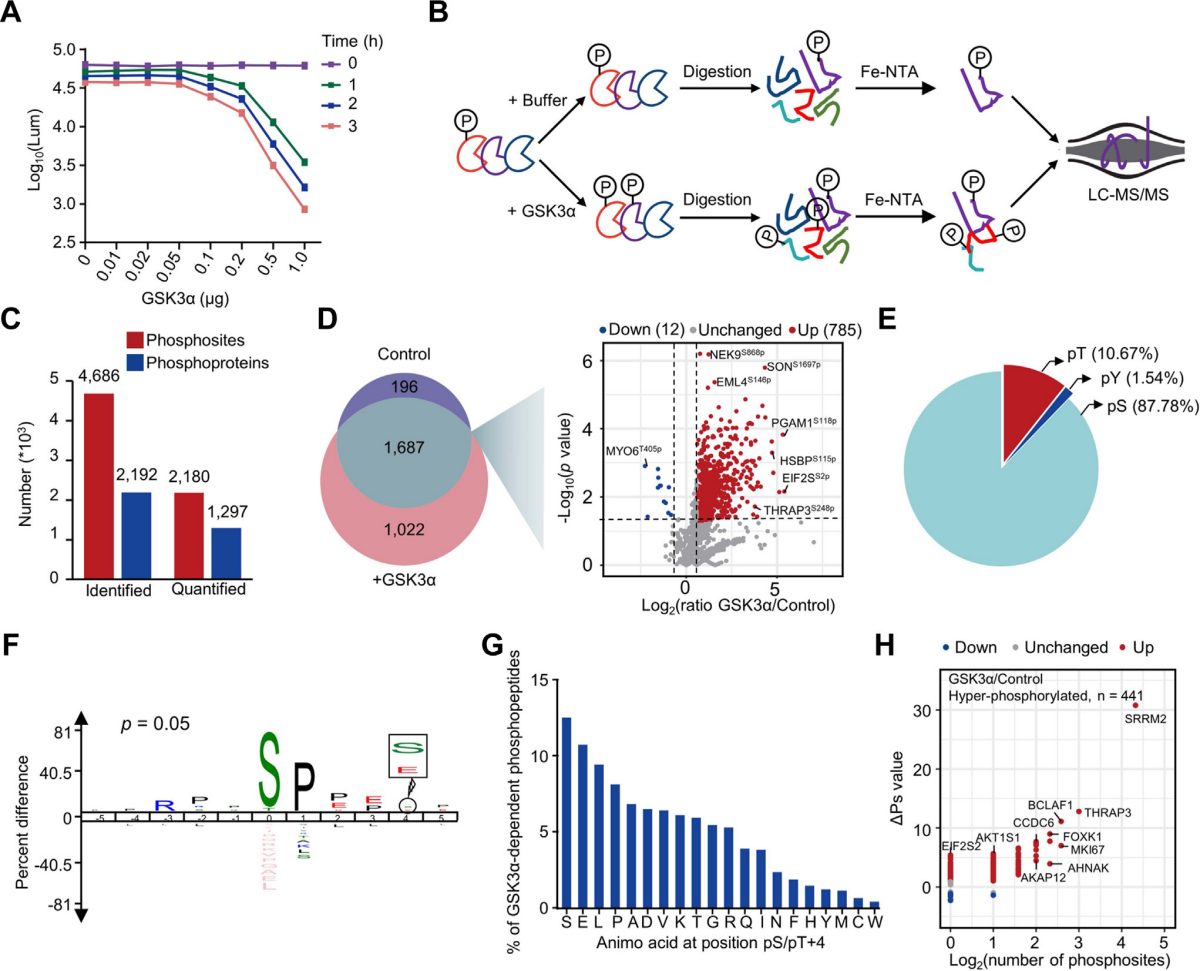
**Profiling *in vitro* phospho-substrates of GSK3α**. *A*, measurement of recombinant GSK3α activity. Equal amounts of protein extracts were mixed with different amounts of recombinant GSK3α and the remaining amount of ATP in the reaction system was detected at different reaction times to measure GSK3α activity. *B*, flowchart of quantitative *in vitro* phosphoproteomics for GSK3α. Recombinant GST-GSK3α was added to the experimental group, while blank buffer was added to the control group. Each group had three repeats. *C*, numbers of identified and quantified phosphosites and their corresponding proteins in the *in vitro* phosphoproteome of GSK3α. *D*, Venn diagram depicting the identified phosphosites in the GSK3α-treated group and control group, and the volcano plot indicates the expression differences of overlapping phosphosites. The *dots in red and blue* indicate the significantly changed phosphosites with foldchanges greater than 1.5 (Student’s *t* test, *p* < 0.05). The phosphosites that are identified in at least 4 of 6 samples or only identified in at least 2 of 3 samples in the control or experimental group are included in the statistics. The phosphosites that were increased 1.5-fold or exclusively identified in the GSK3α-treated group are suggested to be GSK3α-regulated phosphosites *in vitro*. *E*, distribution of GSK3α-regulated phosphosites *in vitro*. *F*, motif analysis of GSK3α-regulated phosphosites *in vitro* using iceLogo. *G*, frequency of amino acids at the +4 position of the GSK3α-regulated phosphosites. *H*, global ΔPs analysis of phosphoproteins using *in vitro* phosphoproteome data of GSK3α. ΔPs means the phosphorylation change value for an individual protein that is calculated by the sum of log2-transformed foldchange of all significantly changed phosphosites between the experimental and control groups (Student’s *t* test, *p* < 0.05). *Blue* represents ΔPs < −1, and *red* represents ΔPs >1. PCA, Principal component analysis.

To clarify the downstream phospho-signals regulated by GSK3α, we next performed *in vitro* and *in vivo* phosphoproteomic analyses. Although a number of GSK3 phospho-substrates have been reported (28), the substrates specifically regulated by GSK3α remain unknown (29). To identify the phospho-substrates of GSK3α *in vitro*, recombinant hGSK3α was expressed in *E. coli.* expression system and purified. The *in vitro* activity of GST-GSK3α was tested by measuring the consumption of ATP, which indicated the good enzymatic activity of recombinant GST-GSK3α (Fig. 3*A*). Then, the purified GST-GSK3α and ATP were incubated with the extracted whole cell lysate. After tryptic digestion, the phosphopeptides were enriched with Fe-NTA agarose beads and analyzed by MS (Fig. 3*B*). In total, we identified 1236 phosphosites increased by 1.5-fold from 843 proteins in response to GST-GSK3α treatment (localization probability of ≥0.75 and Student’s *t* test, *p* < 0.05. 451 phosphosites only identified upon GSK3α treatment were not displayed in the volcano plot.) (Fig. 3, *C* and *D* and supplemental Table S3). The distribution analysis showed that most phosphosites regulated by GSK3α *in vitro* mainly occurred on serine residues (Fig. 3*E*). Motif analysis of GSK3α-mediated phosphosites showed that proline residues were preferred at the +1 and +2 positions (Fig. 3*F*). Approximately 13% of phosphosites might be primed by serine phosphorylation at the +4 position (Fig. 3*G*) (29). In addition, based on a reported algorithm (30), global phosphorylation state change (ΔPs) analysis of phosphoproteins was performed. The phosphorylation state of 441 proteins, including SRRM2, THRAP3, MKI67, BCLAF1, and AHNAK, was significantly increased upon GSK3α treatment (hyperphosphorylated, ΔPs  >  1.0) (Fig. 3*H*).

To compare the phospho-substrates of GSK3α with those of GSK3β *in vitro*, we then carried out the kinase reaction with active recombinant GST-GSK3β *in vitro* in duplicate (supplemental Fig. S5*A*). Pearson’s correlation coefficient of the phosphoproteome between the two repeats was 0.683 (*p* < 0.0001) (supplemental Fig. S5*B*). A total of 571 phosphosites, which had ratio (GSK3β/Control) > 1.5 in duplicate or were only identified in both GSK3β-treated groups, were the most reliable phospho-substrates of GSK3β *in vitro* (supplemental Fig. S5*C* and supplemental Table S3). Motif analysis of GSK3β-mediated phosphosites showed that GSK3β preferred to phosphorylate the threonine residues compared with GSK3α (supplemental Figs. S5*D* and 3*F*). Moreover, proline residues were preferred at the +1 positions of the phosphosites and some phosphorylation events were also primed by serine phosphorylation at the +4 position (supplemental Fig. S5*D*). Unexpectedly, only 49 phosphosites were common substrates of GSK3α and GSK3β *in vitro*, indicating the huge difference between GSK3α and GSK3β as kinases (supplemental Fig. S5*E*). Collectively, these *in vitro* enzymatic results suggest significant functional differences between GSK3β and GSK3α as kinases.

### Identification of GSK3α-Mediated Phospho-Substrates in Colon Cancer Cell Lines

**Fig. 4:**
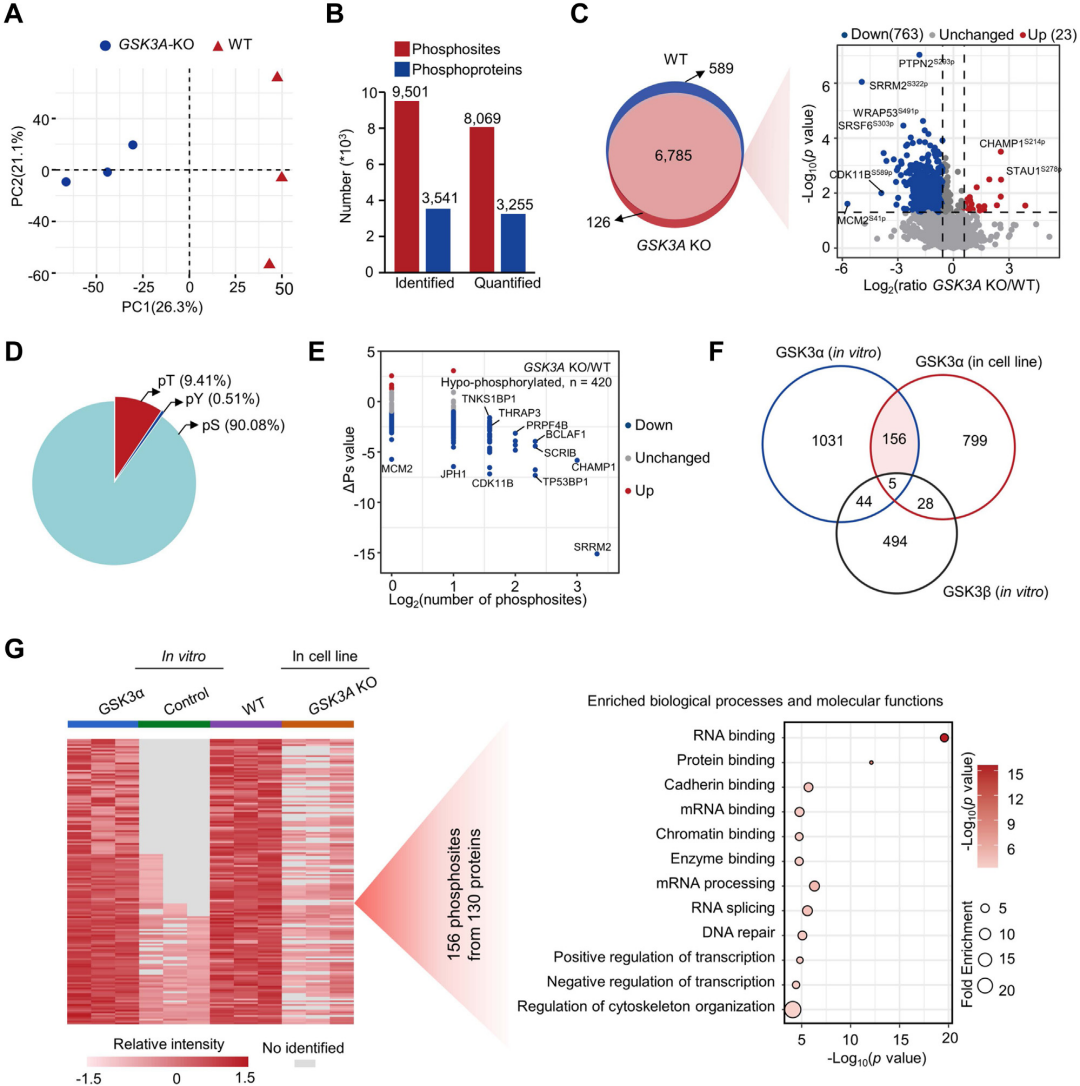
**Profiling the cellular phospho-substrates of GSK3α**. *A*, PCA of the control and experimental groups based on the phosphoproteome data. The control and experimental groups were WT and *GSK3A*-KO HCT116 cell lines, respectively. Each group had three repeats. *B*, numbers of identified and quantified phosphosites and their corresponding phosphoproteins in the phosphoproteomes of *GSK3A*-KO and WT cell lines. *C*, Venn diagram depicting the identified phosphosites in the *GSK3A*-KO and WT groups, and the volcano plot indicates the expression differences of overlapping phosphosites between *the GSK3A*-KO and WT groups. *Red and blue dots* indicate the significantly changed phosphosites with foldchanges greater than 1.5 (Student’s *t* test, *p* < 0.05). The phosphosites that are identified in at least 4 of 6 samples or only identified in at least 2 of 3 samples in the *GSK3A*-KO or WT group are included in the statistics. The phosphosites that were increased 1.5-fold or exclusively identified in the WT group are suggested as GSK3α-regulated phosphosites in cells. *D*, distribution of GSK3α-regulated phosphosites in cells. *E*, global ΔPs analysis of phosphoproteins using the phosphoproteome data of *GSK3A*-KO and WT cell lines. ΔPs indicates the phosphorylation change value for an individual protein that is calculated by the sum of the log2-transformed foldchange of all significantly changed phosphosites between the *GSK3A*-KO and WT groups (Student’s *t* test, *p* < 0.05). *Blue* represents ΔPs < −1, and *red* represents ΔPs >1. *F*, overlap analysis of phosphosites regulated by GSK3α *in vitro* and by GSK3α in cell lines and by GSK3β *in vitro*. *G*, heatmap (*left*) showing the 156 overlapping phosphosites and bubble chart (*right*) showing the enriched pathways using the corresponding 130 phosphoproteins. The filled color of the *circle* represents the *p* value and the size of the *circle* indicates the fold enrichment of each pathway.

*In vitro* phosphorylation assays may not represent *in vivo* regulation. Therefore, by using WT and *GSK3A*-KO colon cancer cell lines, we screened phospho-substrates exclusively regulated by GSK3α in cell lines with three biological repeats. Principal component analysis showed that the WT and *GSK3A*-KO samples could be clearly separated based on the phosphoproteome data (Fig. 4*A*). A total of 9501 phosphosites (localization probability ≥0.75) on 3541 proteins were identified, of which 8069 phosphosites on 3255 proteins were quantified (Fig. 4*B*). Compared with the *GSK3A*-KO group, a total of 988 phosphosites were increased by 1.5-fold in the WT group (Student’s *t* test, *p* < 0.05) (Fig. 4*C* and supplemental Table S4), including 225 phosphosites only identified in the WT group that were not displayed in the volcano plot. Similar to the *in vitro* results, serine modifications accounted for the vast majority (Fig. 4*D*). By analyzing the ΔPs of phosphoproteins, we identified 420 proteins with significantly downregulated total phosphorylation levels in the *GSK3A*-KO group (Fig. 4*E*). Overlapping analysis identified 161 GSK3α-mediated phosphosites *in vitro* and in cell lines, of which 156 were specifically regulated by GSK3α but not GSK3β (Fig. 4*F*). The 156 phosphosites belonged to 130 proteins. Further biological process and molecular function analyses of the 130 phosphoproteins showed that RNA binding, protein binding, cadherin binding, RNA splicing, mRNA processing, DNA repair, and transcriptional regulation were most enriched (Fig. 4*G*). Collectively, we reveal 156 phosphosites specifically regulated by GSK3α.

### The Clinical Significance of GSK3α-Mediated Phosphorylation in Colon Cancer

**Fig. 5:**
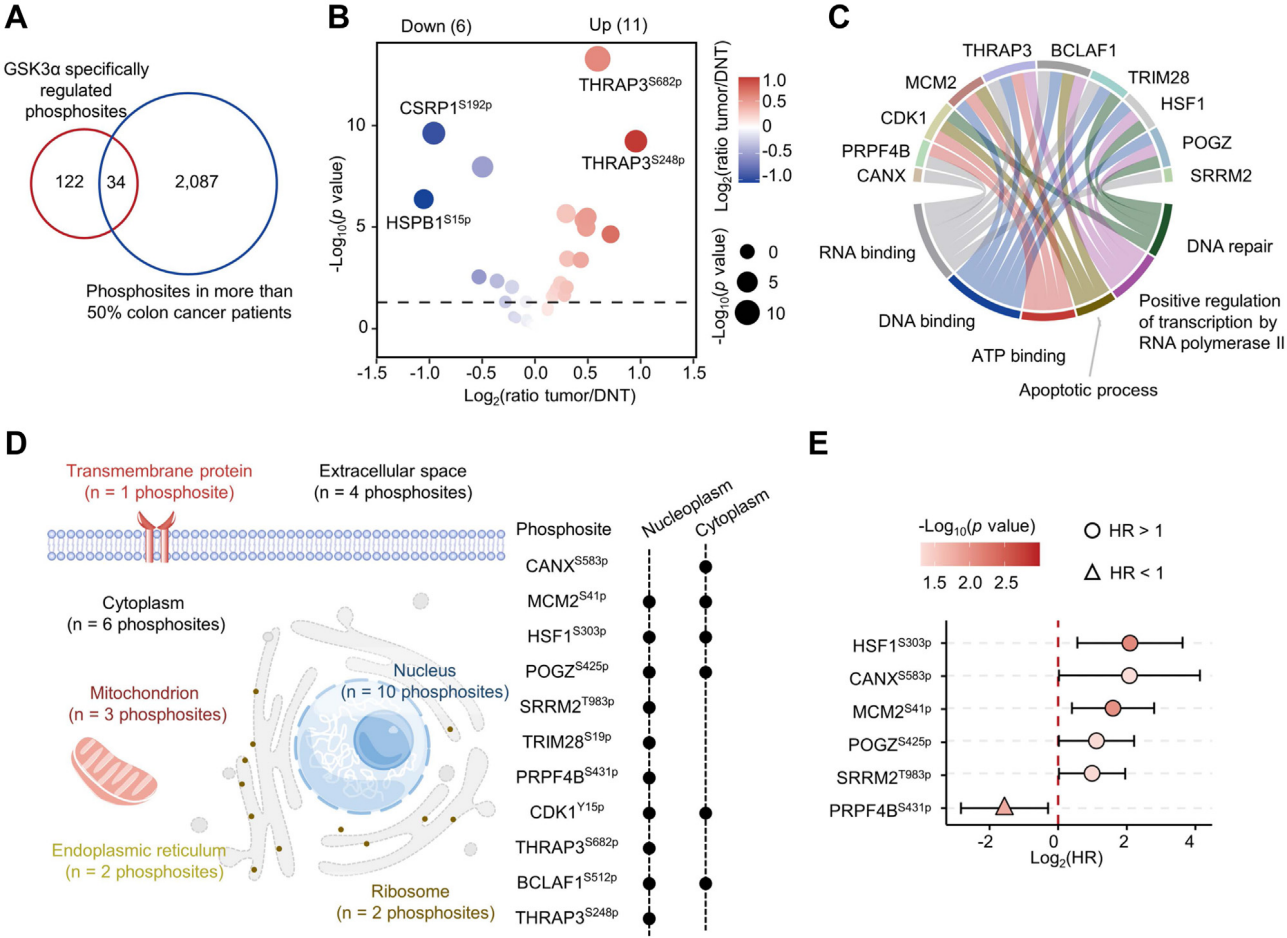
**The clinical significance of GSK3α-regulated phospho-substrates in colon cancer.***A*, overlap analysis of GSK3α-regulated phosphosites and the phosphosites identified in more than 50% of colon cancer patients. *B*, volcano plot displaying the changes in 34 overlapping phosphosites between the tumors and DNTs. The size of the circle indicates the *p* value (Wilcoxon test), and the filled color of the circle indicates the ratio(tumor/DNT). *C*, chord diagrams showing the top 3 Gene Ontology Molecular Function (GO–MF) and Gene Ontology Biological Process (GO–BP) pathways enriched using the 10 phosphoproteins corresponding to the 11 phosphosites. *D*, the cellular localization of the 11 phosphosites significantly upregulated in tumors. *E*, forest plot showing the effect of 6 phosphosites on the prognosis of colon cancer patients. The *p* values were calculated using the Cox proportional hazard model. DNT, distant normal tissue.

To analyze the clinical significance of 156 phosphosites specifically regulated by GSK3α. We analyzed the phosphoproteome data of 70 colon cancer patients (27). Overlapping analysis led to 34 GSK3α-regulated phosphosites that were identified in more than 50% of tumors (Fig. 5*A*). We found that 11 out of the 34 phosphosites, such as THRAP3^S682p^ and THRAP3^S248p^, were increased in tumors (ratio [T/DNT] >1.2 and Wilcoxon test, *p* < 0.05) (Fig. 5*B* and supplemental Table S5). The 11 phosphosites were derived from 10 phosphoproteins, and localization analysis of 10 phosphoproteins showed that these proteins mainly localized in the nucleus (Fig. 5*D*) and were involved in DNA repair, DNA binding, RNA binding, ATP binding, apoptotic process, and positive regulation of transcription by RNA polymerase II (Fig. 5*C*). Survival analysis showed that high levels of 5 phosphosites, including HSF1^S303p^, CANX^S583p^, MCM2^S41p^, POGZ^S425p^, and SRRM2^T983p,^ were significantly correlated with poor prognosis, while high levels of PRPF4B^S431p^ predicted good prognosis (Fig. 5*E*).

### The Potential Roles of THRAP3^S248^ Phosphorylation

**Fig. 6:**
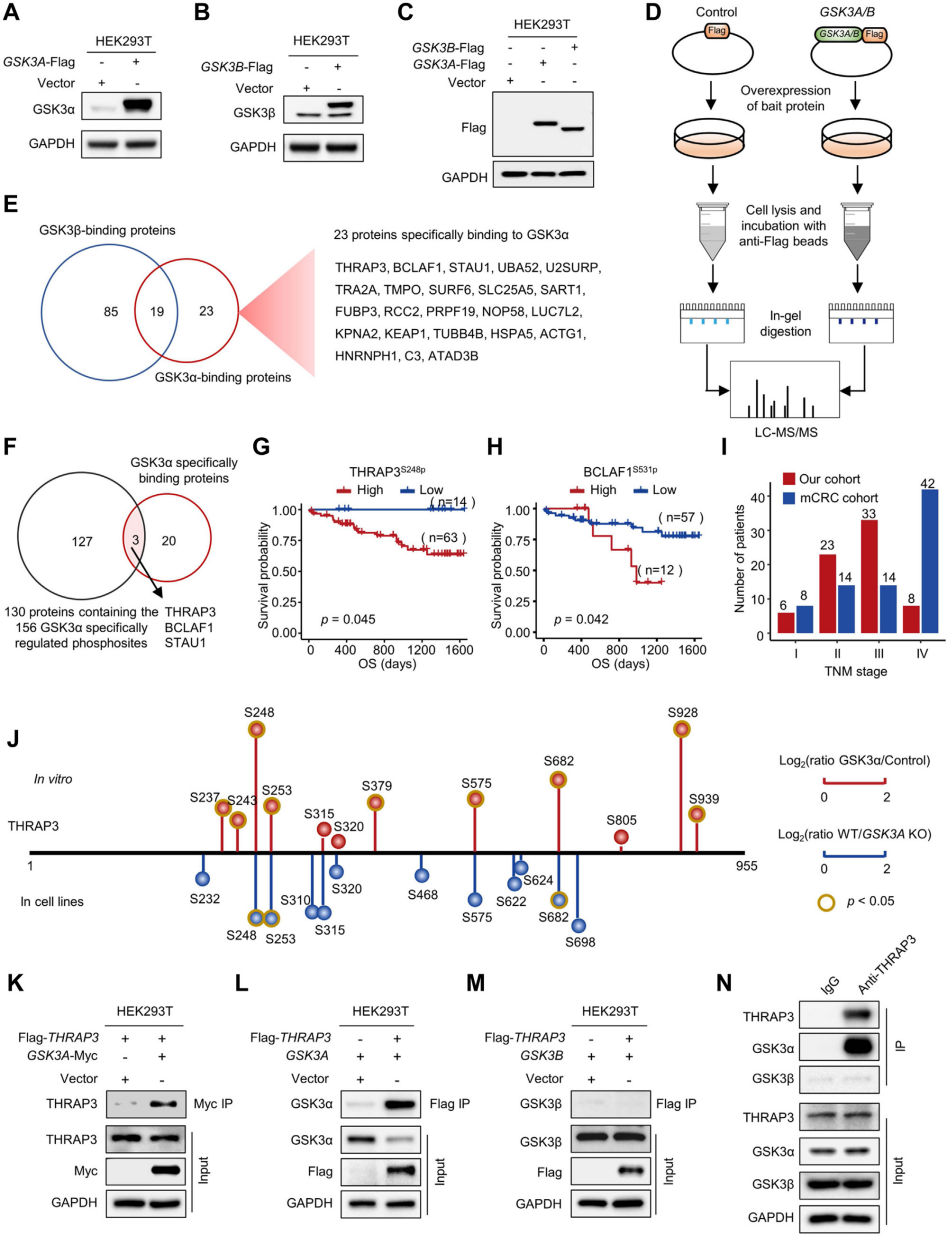
**GSK3α but not GSK3β specifically binds to THRAP3.***A*–*C*, Western blotting analysis of the overexpression of the bait proteins GSK3α-Flag and GSK3β-Flag. *D*, flowchart for the identification of GSK3α- and GSK3β-interacting proteins by affinity purification and MS analysis. *E*, Venn diagram showing the overlap between GSK3α-binding proteins (ratio (GSK3α/Control) >1.5, Student’s *t* test, *p* < 0.05) and GSK3β-binding proteins (ratio (GSK3β/Control) >1.5, Student’s *t* test, *p* < 0.05). *F*, Venn diagram showing the overlap between the 23 GSK3α-specific binding proteins and the 130 proteins containing the 156 phosphosites specifically regulated by GSK3α. *G* and *H*, survival analysis of colon cancer patients based on the levels of THRAP3^S248p^ (*G*) and BCLAF1^S531p^ (*H*) using the phosphoproteome data of the mCRC cohort. *I*, statistical analysis of colon cancer patients in different TNM stages in our cohort and mCRC cohort. *J*, eighteen phosphosites on THRAP3 identified *in vitro* and in the HCT116 cell line. The *red balls* indicate the phosphosites on THRAP3 upregulated after adding GSK3α *in vitro*. The *blue balls* indicate the decreased phosphosites on THRAP3 after knocking out *GSK3A*. The length of the *vertical line* indicates the relative foldchange. The longer the line is, the greater the change. The *dark yellow* outer circle indicates significant changes at that site (Student’s *t* test, *p* < 0.05). *K*, immunoblots of the binding proteins of GSK3α-myc using anti-THRAP3 antibody. *L*–*N*, immunoblots of the binding proteins of Flag-THRAP3 using anti-GSK3α (*L*) and anti-GSK3β (*M*) antibodies. *N*, immunoblots of THRAP3 coimmunoprecipitated proteins using anti-GSK3α and anti-GSK3β antibodies. THRAP3 was enriched using an anti-THRAP3 antibody. Enrichment by IgG was used as a control. CRC, Colorectal cancer.

We speculated that of the 156 phosphosites specifically regulated by GSK3αand their corresponding proteins might have different binding affinities to GSK3α. To identify the protein substrates most tightly bound to GSK3α, we overexpressed *GSK3A*-Flag and *GSK3B*-Flag vectors in the 293T cell line (Fig. 6, *A*–*C*) and performed pull-down experiments with an anti-Flag antibody (31). The binding proteins of GSK3α and GSK3β were analyzed by MS (Fig. 6*D*). As a result, 23 proteins specifically bound to GSK3α but not GSK3β (Fig. 6*E* and supplemental Table S6). Of the 23 proteins, 3 proteins, including THRAP3, BCLAF1, and STAU1, were phosphoproteins specifically regulated by GSK3α (Fig. 6*F*). Notably, of the 156 phosphosites specifically regulated by GSK3α, 9 phosphosites were derived from THRAP3, BCLAF1, and STAU1. Among the 9 phosphosites, 4 sites, including THRAP3^S248p^, THRAP3^S682p^, BCLAF1^S512p^, and BCLAF1^S531p^, were also identified in the phosphoproteomics analysis of paired tissues from 70 colon cancer patients (supplemental Table S5 and Fig. 5*A*). However, none of them showed a significant correlation with OS in our cohort of 70 colon cancer patients. Interestingly, of the 4 phosphosites, the levels of THRAP3^S248p^ and BCLAF1^S531p^ were significantly correlated with OS in another colon cancer cohort termed the mCRC cohort (32) (Fig. 6, *G* and *H*), in which more metastatic colon cancer patients were included (Fig. 6*I*).

THRAP3 is a protein with multiple phosphorylation sites. Of the 18 identified phosphosites in this work, phosphorylation at S248, S253, and S682 was specifically mediated by GSK3α *in vitro* and in cell lines (Fig. 6*J*). Because THRAP3^S248p^ but not BCLAF1^S531p^ was significantly increased in tumors compared with DNTs (Fig. 5*B*), the function of THRAP3^S248p^ was further investigated. Based on the evidence from the mCRC cohort (32), we assumed that THRAP3^S248p^ might play a role in the process of tumor metastasis. To reveal the function of THRAP3^S248p^, the binding of THRAP3 to GSK3α was first confirmed by immunoblotting (Fig. 6*K*). Retro-pull-down of GSK3α and GSK3β by THRAP3 was also performed. The results showed that GSK3α but not GSK3β could be pulled down by THRAP3 (Fig. 6, *L* and *M*). To exclude the influence of the tag on the pull-down results, we used an anti-THRAP3 antibody to enrich endogenous GSK3. As expected, GSK3α, but not GSK3β, was specifically enriched by the anti-THRAP3 antibody (Fig. 6*N*).

**Fig. 7:**
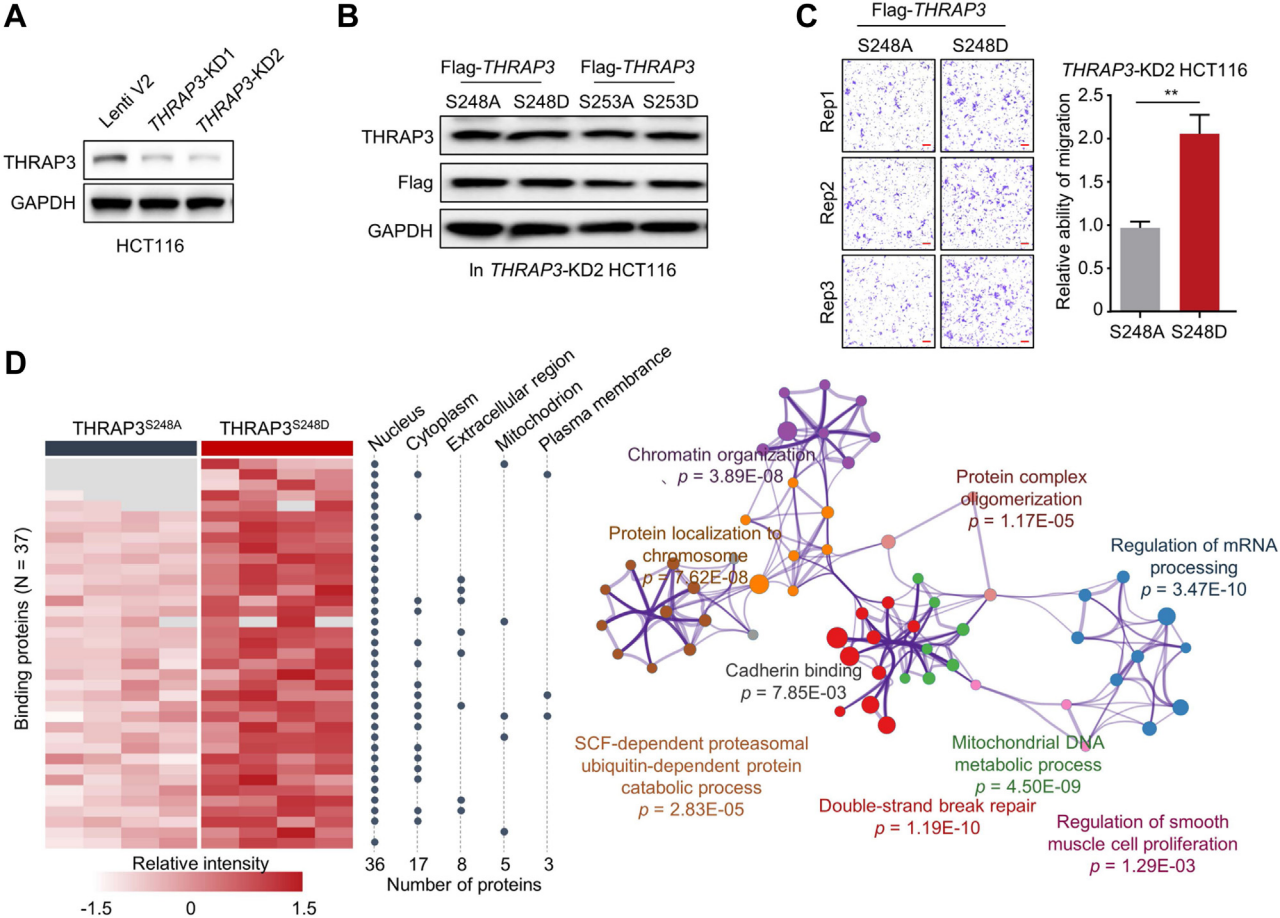
**THRAP3**^**S248p**^**increases cancer cell migration and its binding affinity to proteins related to DNA damage repair.***A*, Western blotting analysis of the THRAP3 levels in *THRAP3*-KD HCT116 cells. *B*, Western blotting analysis of the levels of THRAP3^S248A^, THRAP3^S248D^, THRAP3^S253A^, and THRAP3^S253D^ after overexpressing the corresponding vectors. *C*, representative images of the migration assay using *THRAP3*-KD HCT116 cells after overexpressing THRAP3^S248A^ and THRAP3^S248D^. The scale bar represents 100 μm. Each assay was repeated three times. The *p* value was calculated using paired *t* test. ∗∗*p* < 0.01. *D*, thirty-seven differentially bound proteins between THRAP3^S248A^ and THRAP3^S248D^ mutants and their subcellular localization (*left*). The enriched pathways of the 37 proteins by GO analysis are shown on the *right*.

Because the S248 and S253 residues of THRAP3 are very close in the sequence, the function of THRAP3^S253p^ in colon cancer was also investigated for comparison. To disclose the functions of THRAP3^S248p^ and THRAP3^S253p^ in colon cancer, we silenced the *THRAP3* gene in the HCT116 cell line (Fig. 7*A*) and then overexpressed two pairs of THRAP3 mutants, Flag-THRAP3^S248A^ and Flag-THRAP3^S248D^, as well as Flag-THRAP3^S253A^ and Flag-THRAP3^S253D^, in the *THRAP3*-KD HCT116 cell line (Fig. 7*B*). Of these mutants, Flag-THRAP3^S248A^ and Flag-THRAP3^S253A^ mimicked the effect of nonphosphorylation, and Flag-THRAP3^S248D^ and Flag-THRAP3^S253D^ mimicked the effect of phosphorylation. As a result, we did not observe a significant difference in clonogenic growth capability between cancer cell lines overexpressing THRAP3^S248A^ and THRAP3^S248D^ mutants (supplemental Fig. S6*A*). However, compared with THRAP3^S248A^, THRAP3^S248D^ mimicking phosphorylation was able to enhance the migration of HCT116 cells (Fig. 7*C*), consistent with our assumption that THRAP3^S248p^ is closely linked to tumor metastasis. It should be mentioned that both THRAP3^S253D^ and THRAP3^S248/253D^ had no effect on cancer cell migration (supplemental Fig. S6, *B*–*D*).

We next performed pull-down experiments to identify the THRAP3^S248p^-binding proteins and identified 37 proteins that tended to bind to THRAP3^S248D^ and 0 proteins that tended to bind to THRAP3^S248A^ (Fig. 7*D* and supplemental Table S7). Enrichment analysis showed that these proteins were involved in many biological pathways, such as double-strand break repair (*p* = 1.19E-10) and cadherin binding (*p* = 7.85E-03) (Fig. 7*D*). In contrast, 17 and 25 proteins preferred to bind to THRAP3^S253D^ and THRAP3^S253A^, respectively (supplemental Fig. S6*E* and supplemental Table S7). The functions of THRAP3^S253D^- and THRAP3^S253A^-binding proteins were different from those of THRAP3^S248D^-binding proteins (supplemental Fig. S6*E*). Collectively, the above results show the potential roles of THRAP3^S248p^ in regulating colon cancer cell migration and DDR.

## Discussion

In summary, we investigated the kinase functions of GSK3α and its specific phospho-substrates in colon cancer. Several new achievements have been gained. First, we demonstrated that high expression of GSK3α, but not GSK3β, was significantly correlated with unfavorable outcomes in colon cancer patients from four independent cohorts. Second, we identified 156 phospho-substrates specifically regulated by GSK3α, but not GSK3β, *in vitro* and in colon cancer cell lines. Furthermore, a number of phospho-substrates of GSK3α have been identified in tissues from colon cancer patients and some of them show a significant correlation with the OS of colon cancer patients. Third, we investigated the potential roles of THRAP3^S248p^, a phosphosite regulated by GSK3α, in colon cancer and showed that THRAP3^S248p^ may function in the regulation of colon cancer cell migration and DDR. Collectively, this work not only discloses the specific function of GSK3α as a kinase but also suggests GSK3α as a promising therapeutic target for colon cancer.

GSK3α and GSK3β have almost identical sequences in the kinase domain but are distinct at the C terminus and N terminus. All available inhibitors are known to inhibit both isoforms but many studies erroneously attribute the inhibitory effect to specific inhibition of GSK3β while ignoring effects on GSK3α (33). In addition, clinical trials of dual GSK3α/β inhibitors have not proven successful (4). We showed that GSK3α and GSK3β differ greatly in their binding proteins (Fig. 6*E*) and phospho-substrates (Fig. 4*F*) in colon cancer cells, although we cannot exclude the possibility that the phospho-substrates of GSK3α and GSK3β may differ in different cell types. GSK3α was more closely associated with colon cancer progression and unfavorable outcomes than GSK3β (Figs. 1, *A* and *B* and 1*F*). Therefore, we assume that specific inhibition or targeted degradation of GSK3α by proteolysis-targeting chimeras may be an effective strategy for the treatment of colon cancer (34, 35).

A number of known GSK3 phospho-substrates have been more accurately identified as substrates of GSK3β or GSK3α. For example, we have confirmed that DYPSL2^T509p^ (36, 37, 38), UNG^T60p^ (39), PHF6^S155p^ (29), and RPS14^T133p^ (29) are common substrates of GSK3α and GSK3β. FOXK1^S428p^ (40) and PKM^T328p^ (41) are specific substrates of GSK3α and GSK3β, respectively. In addition, we also obtained some results that are inconsistent with previous reports. Some phosphosites, such as DPYSL3^T509p^ (38), MAPRE1^S155p^ (42), BCLAF1^S531p^ (43), and RPTOR^S859p^ (44), are GSK3α-specific substrates based on our work but these phosphosites were incorrectly assigned to GSK3β substrates in the PhosphoSitePlus database (www.phosphosite.org). The reason for these results may be that the investigators used GSK3 inhibitors but classified the affected phosphosites as substrates of GSK3β. In addition, we also found that some phosphosites previously clearly reported as substrates of GSK3β can also be regulated by GSK3α *in vitro*, such as SRF^S224p^ (45) and SOX9^T236p^ (46). The different cell types used may also be an important reason for these results. Collectively, these results indicate that clarification of the substrate specificity of GSK3α and GSK3β may help to obtain the correct conclusions.

Comparing the *in vitro* phosphoproteome data (Fig. 3*C*) to the cell line phosphoproteome data (Fig. 4*B*), it was observed that many more phosphosites and phosphoproteins were identified in the cell lines, indicating that the phosphorylation events are more complex in cells than *in vitro*. Moreover, secondary phosphorylation reactions in the cells can further increase the complexity. For example, some identified phosphoproteins, which are substrates of GSK3α, are also kinases, such as CDK1, CDK11B, and MAPK1. GSK3α-mediated phosphorylation of these kinases may further phosphorylate downstream protein substrates, which are substrates of downstream kinases rather than direct substrates of GSK3α. Notably, the number of phosphosites regulated by GSK3α was greater in the *in vitro* enzymatic assays (Fig. 4*F*). Previous studies have suggested that kinases may be less specific *in vitro* than *in vivo* (47), which could lead to more differential phosphorylation reactions *in vitro*, consistent with our observations.

THRAP3, also called TRAP150 and BCLAF2, is a member of the BRCA1-interacting RNA splicing complex involved in promoting pre-mRNA splicing and subsequent transcript stability in response to DNA damage (48, 49). THRAP3 deficiency or site mutations increase sensitivity to DNA damaging agents (50), which induce ATR-associated phosphorylation on multiple serine residues of THRAP3, such as S210, S211, S399, S406, and S408 (51). THRAP3 also regulates mRNA export to the cytoplasm and many of the transcripts exported by THRAP3 are associated with DDR (50). In this work, we showed that THRAP3^S248p^, THRAP3^S253p^, and THRAP3^S682p^ are GSK3α-specific phosphosites, and THRAP3^S248p^ may also be linked to the DDR process (Fig. 7*D*). In addition, we also revealed the roles of THRAP3^S248p^ in regulating cancer cell migration. Although THRAP3^S248p^ and THRAP3^S253p^ are very close in sequence, no synergistic effect in regulating cancer cell migration was observed (supplemental Fig. S6*D*).

In addition to THRAP3, we also identified many novel phosphosites specifically regulated by GSK3α. However, most of their functions are unknown. For example, BCLAF1 is a BCL-2-associated transcriptional repressor and can induce cell apoptosis. SRSF10 is a key regulator of BCLAF1 pre-mRNA splicing. High SRSF10 expression in CRC samples increases the inclusion of BCLAF1 exon 5a, which is associated with higher CRC grade (52). Phosphorylation is critical to regulate the functions of BCLAF1. BCLAF1^S290p^ is also a known phosphosite involved in the regulation of the DNA damage response (53). DNA-PKC-mediated phosphorylation of BCLAF1^Y150^ and BCLAF1^S151^ is located in an RS domain and drives BCLAF1 to the nuclear envelope (54). GSK3 has been predicted to be the kinase of BCLAF1 but it is not clear whether GSK3α or GSK3β plays a role (43). Moreover, it is also unclear which sites on BCLAF1 are regulated by GSK3. Here, we confirmed that GSK3α can regulate BCLAF1 phosphorylation and identified five unambiguous phosphosites mediated by GSK3α, which may help to clarify the functions of these phosphosites.

There are still some limitations in this work. First, the roles of GSK3α-regulated phosphosites remain to be disclosed, especially those whose levels are significantly correlated with the OS of colon cancer patients. Second, the question of how THRAP3^S248p^ responds to DNA damage *via* its binding proteins or binding nucleic acids requires further investigation.

## Data Availability

All MS raw data and the output tables have been deposited to the ProteomeXchange Consortium (http://proteomecentral.proteomexchange.org) *via* the iProX partner repository, with the dataset identifiers PXD038081 (the phosphoproteome data of the 70 paired colon cancer tissues) and PXD038830 (other omics data except the phosphoproteome data of the 70 paired colon cancer tissues).The annotated spectra has been deposited to the MS-Viewer with the unique search key for each batch of colon cancer proteomic data provided in the supplemental Table S1, colon cancer phosphoproteomic data provided in the supplemental Table S5, *in vitro* phosphoproteomic data of GSK3α, and GSK3β provided in the supplemental Table S3, in cell lines phosphoproteomic data of GSK3α provided in the supplemental Table S4, coimmunoprecipitation proteomic data of GSK3α and GSK3β provided in the supplemental Table S6, and coimmunoprecipitation proteomic data of THRAP3 mutant (THRAP3^S248A^ and THRAP3^S248D^, THRAP3^S253A^ and THRAP3^S253D^) provided in the supplemental Table S7.

## Supplemental data

This article contains supplemental data.

## Conflict of Interest

There is no competing interest.
