# Supplementary material for: Deciphering the Clinical Significance and Kinase Functions of GSK3α in Colon Cancer by Proteomics and Phosphoproteomics
Source: Mol Cell Proteomics. 2023 Apr 8;22(5):100545. doi: 10.1016/j.mcpro.2023.100545 (PMC10196724; doi:10.1016/j.mcpro.2023.100545)

supplemental Fig. S1

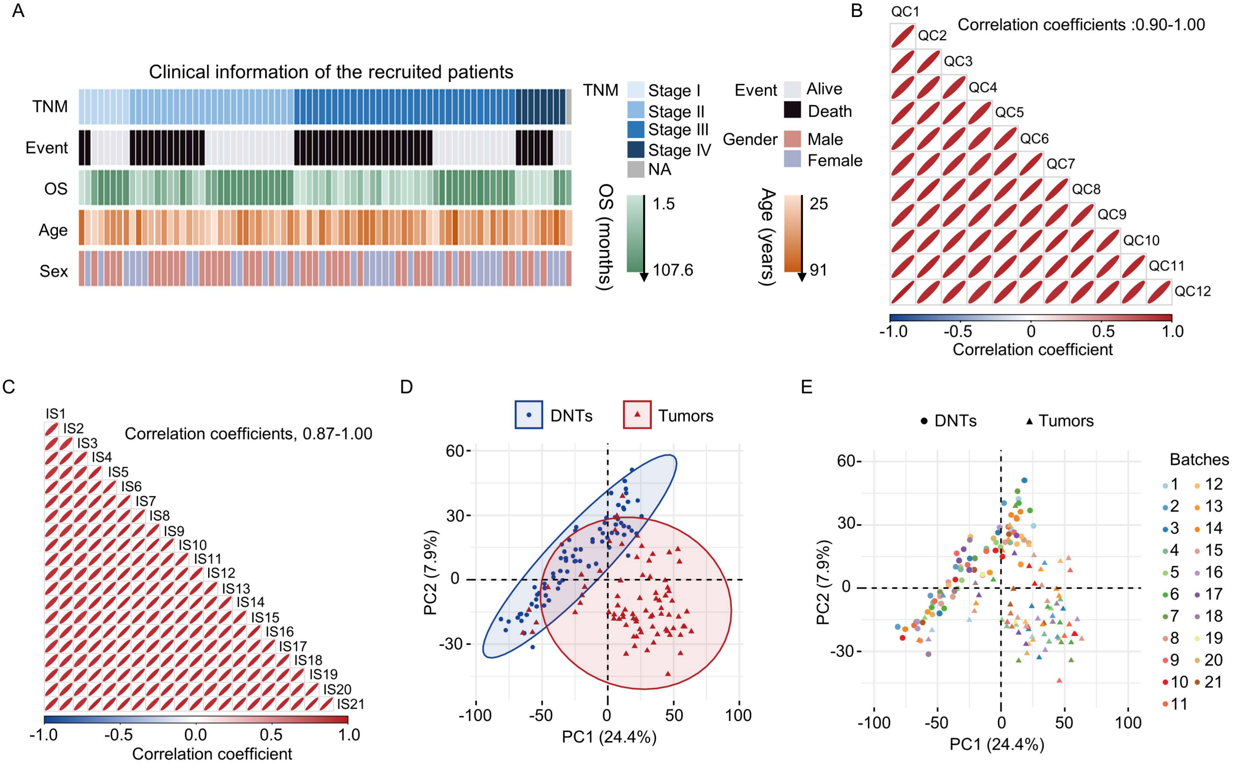

supplemental Fig. S2

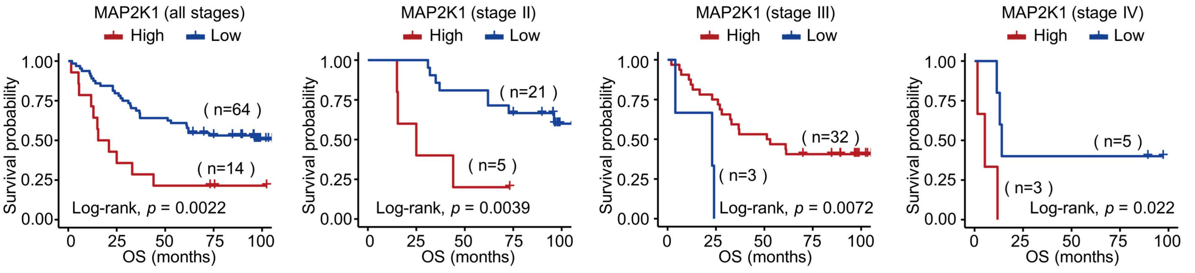

supplemental Fig. S3

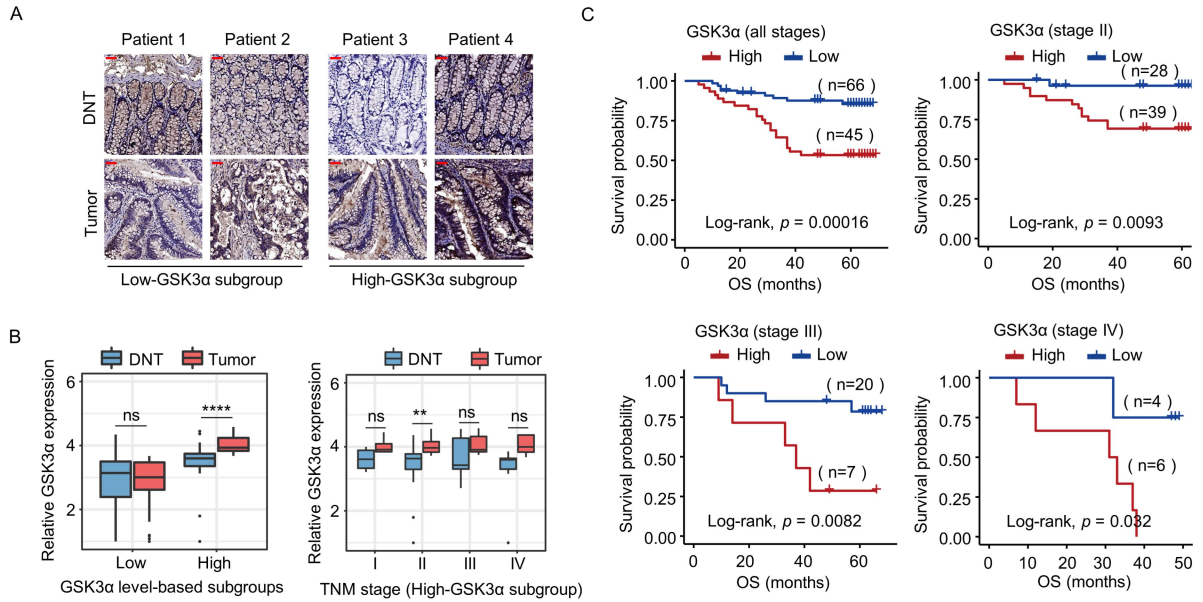

supplemental Fig. S4

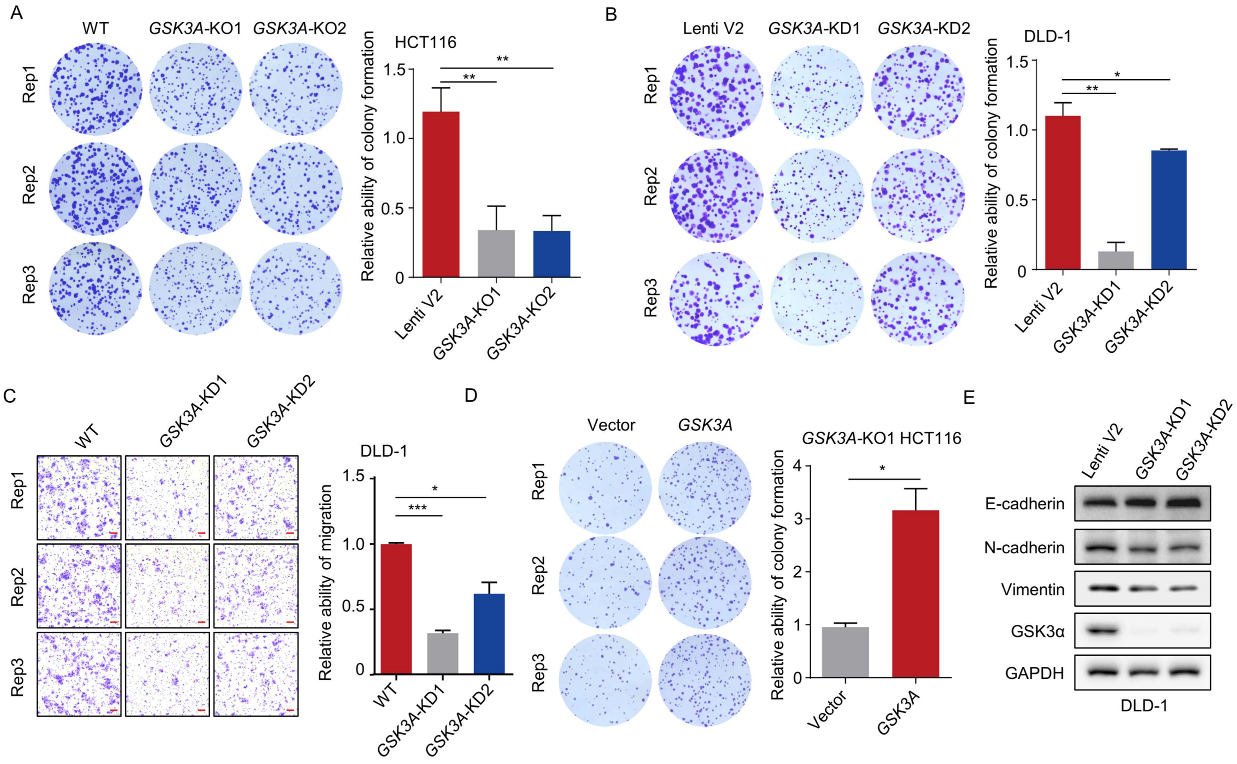

supplemental Fig. S5

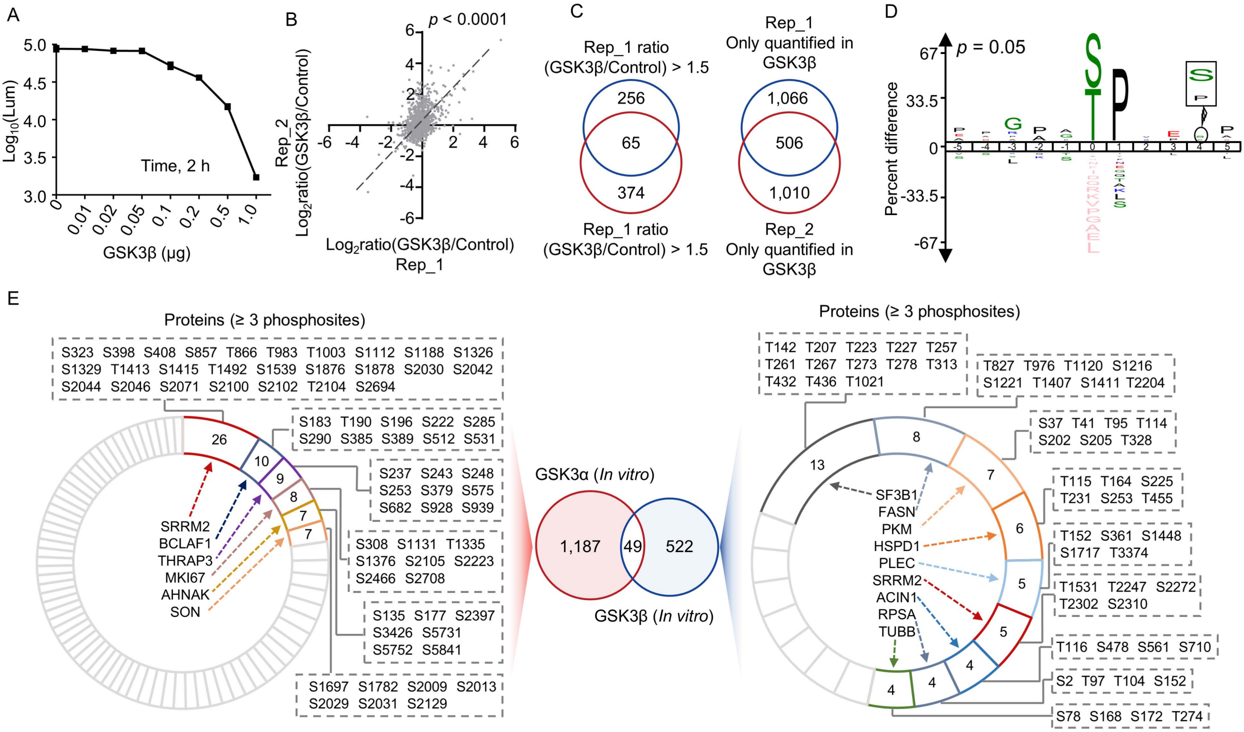

supplemental Fig. S6

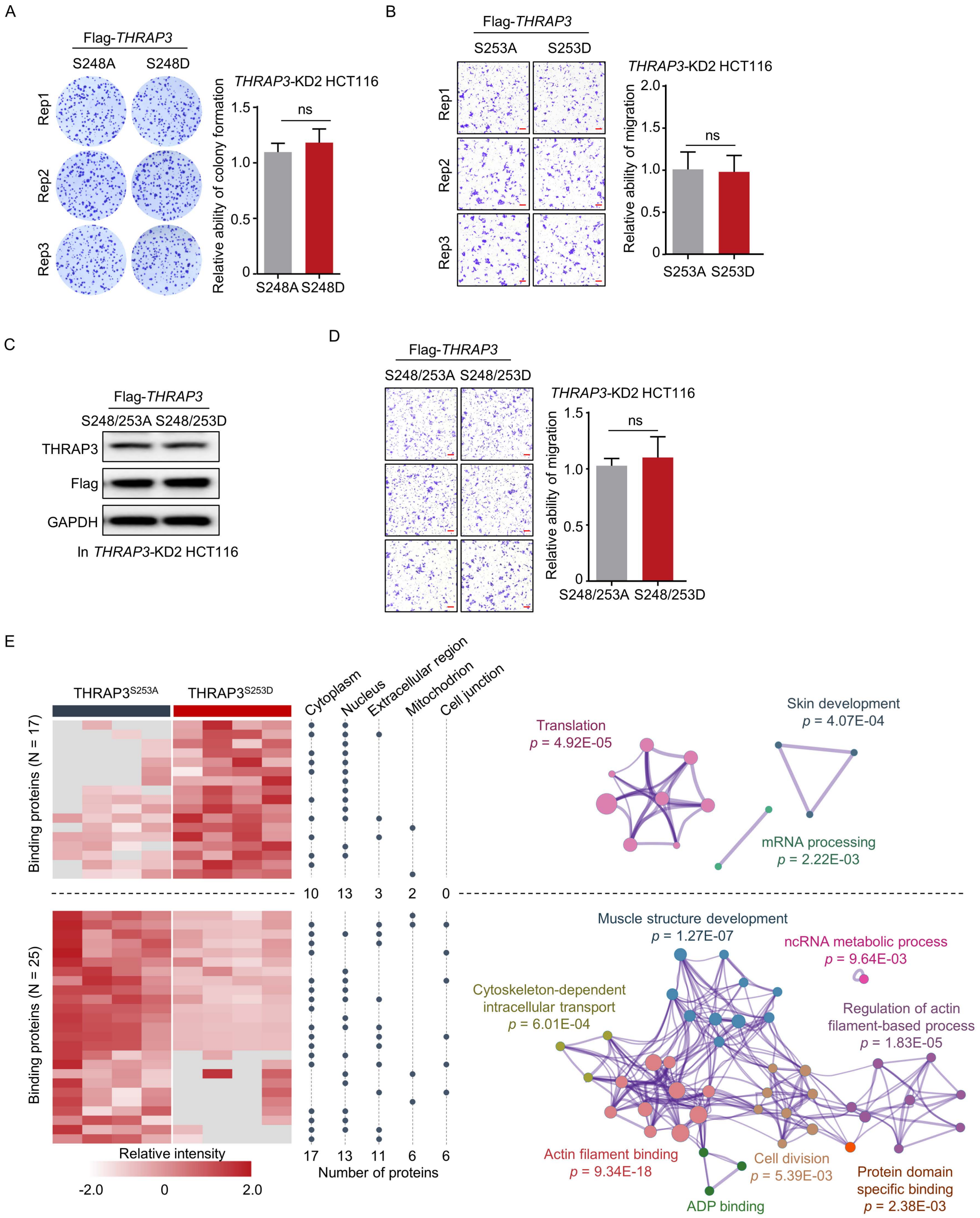

Supplement: Supplemental Figures [file mmc8.pdf]
