# Supplementary material for: Deciphering the Clinical Significance and Kinase Functions of GSK3α in Colon Cancer by Proteomics and Phosphoproteomics
Source: Mol Cell Proteomics. 2023 Apr 8;22(5):100545. doi: 10.1016/j.mcpro.2023.100545 (PMC10196724; doi:10.1016/j.mcpro.2023.100545)
Supplement: Supplemental information [file mmc9.docx]

*Supplemental information*

**Deciphering the clinical significance and kinase functions of GSK3α in colon cancer by proteomics and phosphoproteomics**

Li Gao,^1,†^ Ying Lu,^1,†^ Hai-Ning Chen,^2,†^ Zhigui Li,^3,†^ Meng Hu,^1,†^ Rou Zhang,^1^ Xiuxuan Wang,^1^ Zhiqiang, Xu,^1^ Yanqiu Gong,^1^ Rui Wang,^4^ Dan Du,^4^ Shan Hai,^1^ Shuangqing Li,^1^ Dan Su,^1^ Yuan Li,^5^ Heng Xu,^1^ Zong-Guang Zhou,^2,5^ Lunzhi Dai^1,^*

^1^National Clinical Research Center for Geriatrics and General Practice Ward/International Medical Center Ward, General Practice Medical Center, State Key Laboratory of Biotherapy, West China Hospital, Sichuan University, Chengdu, 610041, China.

^2^Colorectal Cancer Center, Department of General Surgery, West China Hospital, Sichuan University, Chengdu, 610041, China.

^3^Department of Gastrointestinal Surgery, West China Hospital, Sichuan University, Chengdu, 610041, China.

^4^Advanced Mass Spectrometry Center, Research Core Facility, Frontiers Science Center for Disease-related Molecular Network, West China Hospital, Sichuan University, Chengdu, 610041, China.

^5^Institute of Digestive Surgery, West China Hospital, Sichuan University, Chengdu, 610041, China.

^†^These authors contributed equally to this work.

*Correspondence: lunzhi.dai@scu.edu.cn (Dr. Lunzhi Dai)

**Supplemental Figure legends**

**supplemental F_IG_. S1. Quality control (QC) assessment of the proteome data.**

*A*, Illustration of clinical information of the 78 colon cancer patients, including TNM stages (8 cases for stage I, 26 cases for stage II, 35 cases for stage III, 8 cases for stage IV and 1 case without TNM stage information), events (36 alive cases and 42 death cases), survival time to the date of last contact (1.5 months to 107.56 months), age (25 years to 91 years) and sex (35 female and 43 male). *B*, Pearson’s correlation analysis of the 12 QC samples. The correlation coefficients range from 0.90 to 1.00. *C*, Pearson’s correlation analysis of the common reference samples in 21 batches. The correlation coefficients range from 0.87 to 1.00. *D*, Principal component analysis (PCA) of tumors and DNTs using the proteome data. *E*, PCA of different batches of TMT-labeled samples using the proteome data.

**supplemental F_IG_. S2. Correlation analysis between the levels of MAP2K1 and overall survival of colon cancer patients.**

Survival analysis of colon cancer patients with different MAP2K1 expression in tumors of all TNM stages or in tumors of a specific TNM stage. *p* values were calculated by the log-rank test, and the best cutoff was applied.

**supplemental F_IG_. S3. Further validation of the clinical significance of GSK3α**

*A*, Representative immunohistochemistry (IHC) images for the expression of GSK3α in DNTs and tumors in the low-GSK3α and high-GSK3α subgroups. Scale bar, 50 μm. The low-GSK3α and high-GSK3α subgroups were divided based on the best cutoff of OS, as shown in supplemental Fig. S3*C*. *B*, Relative GSK3α expression between tumors and DNTs in the low-GSK3α and high-GSK3α subgroups and the relative GSK3α expression in four TNM stages in the high-GSK3α subgroup. The significance of differences between different groups was calculated by the Wilcoxon test, ** *p* < 0.01, **** *p* < 0.0001. *C*, Survival analysis of colon cancer patients with different levels of GSK3α in all tumors or in tumors of different TNM stages (stage II, stage III and stage IV) based on IHC intensity. The *p* value was calculated by the log-rank test, and the best cutoff was selected.

**supplemental F_IG_. S4. The clonal formation ability of colon cancer cell lines in response to *GSK3A* silencing and overexpression.**

*A*, Clonal formation assays of WT and *GSK3A*-KO HCT116 cells. *B*, Clonal formation assays of negative control and *GSK3A*-KD DLD-1 cells. *C*, Representative images of DLD-1 colon cancer cell line migration after knocking down *GSK3A*. Scale bar, 100 μm. *D*, Clonal formation assays of *GSK3A*-KO HCT116 cells after re-expressing *GSK3A* and vector. *E*, Immunoblots of GSK3α, E-cadherin, N-cadherin and vimentin in DLD-1 cells in response to *GSK3A* silencing. For supplemental Fig. S4*A*-*D*. Each experiment had three repeats, and a paired *t* test was used for the indicated comparison, * *p* < 0.05, ** *p* < 0.01.

**supplemental F_IG_. S5. *In vitro* phospho-substrates of GSK3β.**

*A*, Activity detection of recombinant GST-GSK3β with three repeats. *B*, Pearson’s correlation analysis of the duplicated *in vitro* phosphoproteome mediated by GSK3β. *C*, Overlapping analysis reveals the phosphosites regulated by GSK3β *in vitro* in duplicate. Phosphosites with ratio(GSK3β/Control) greater than 1.5-fold (left) or only quantified in GSK3β treatment (right) were applied for overlapping analysis. *D*, Sequence motif analysis of the phosphosites regulated by GSK3β *in vitro* by iceLogo. *E*, Venn diagram depicting the overlapping phospho-substrates of GSK3α and GSK3β *in vitro*, and the circular plot displays the phospho-substates that are specifically regulated by GSK3α or GSK3β. Proteins with 3 or more phosphosites regulated by GSK3α or GSK3β *in vitro* are shown.

**supplemental F_IG_. S6. The potential functions of THRAP3^S248p^ and THRAP3^S253p^.**

*A*, Colony formation ability after re-expressing THRAP3^S248A^ and THRAP3^S248D^ in *THRAP3*-KD2 HCT116 cells. *B*, Representative images of *THRAP3*-KD2 HCT116 cell migration after re-expressing THRAP3^S253A^ and THRAP3^S253D^. Scale bar, 100 μm. *C*, Immunoblots of the THRAP3^S248/253A^ and THRAP3^S248/253D^ mutants in *THRAP3*-KD2 HCT116 cells. *D*, Representative images of *THRAP3*-KD2 HCT116 cell migration after re-expression of THRAP3^S248/253A^ and THRAP3^S248/253D^. Scale bar, 100 μm. *E*, The binding partners of THRAP3^S253A^ and THRAP3^S253D^. The subcellular localization and the enriched pathways of the binding proteins are displayed. For supplemental Figs. S5*A-B* and S5*D*, each experiment had three repeats, and a paired *t* test was used for the indicated comparison.

**Supplemental Table legends**

**supplemental Table S1.** Clinical information of the 78 colon cancer patients and the results for the screen of clinically associated kinases.

**supplemental Table S2.** Information on the colon cancer tissue microarray and the GSK3α intensity of each spot in the microarray.

**supplemental Table S3.** List of phospho-substrates of GSK3α and GSK3β *in vitro*.

**supplemental Table S4.** List of phospho-substrates of GSK3α in cell lines.

**supplemental Table S5.** The clinical significance analysis results of the phosphosites specifically regulated by GSK3α.

**supplemental Table S6.** The binding proteins of GSK3α and GSK3β.

**supplemental Table S7.** The binding proteins of THRAP3 mutants included Flag-THRAP3^S248A^, Flag-THRAP3^S248D^, Flag-THRAP3^S253A^ and Flag-THRAP3^S253D^.
